# Supplementary material for: Adjuvant-dependent impact of inactivated SARS-CoV-2 vaccines during heterologous infection by a SARS-related coronavirus
Source: Nat Commun. 2024 May 3;15:3738. doi: 10.1038/s41467-024-47450-x (PMC11068739; doi:10.1038/s41467-024-47450-x)
Supplement: Supplementary file 1 — Supplementary Information [file 41467_2024_47450_MOESM1_ESM.pdf]

Adjuvant-dependent impact of inactivated SARS-CoV-2 vaccines during heterologous infection by a SARS-related coronavirus

SUPPLEMENTARY INFORMATION

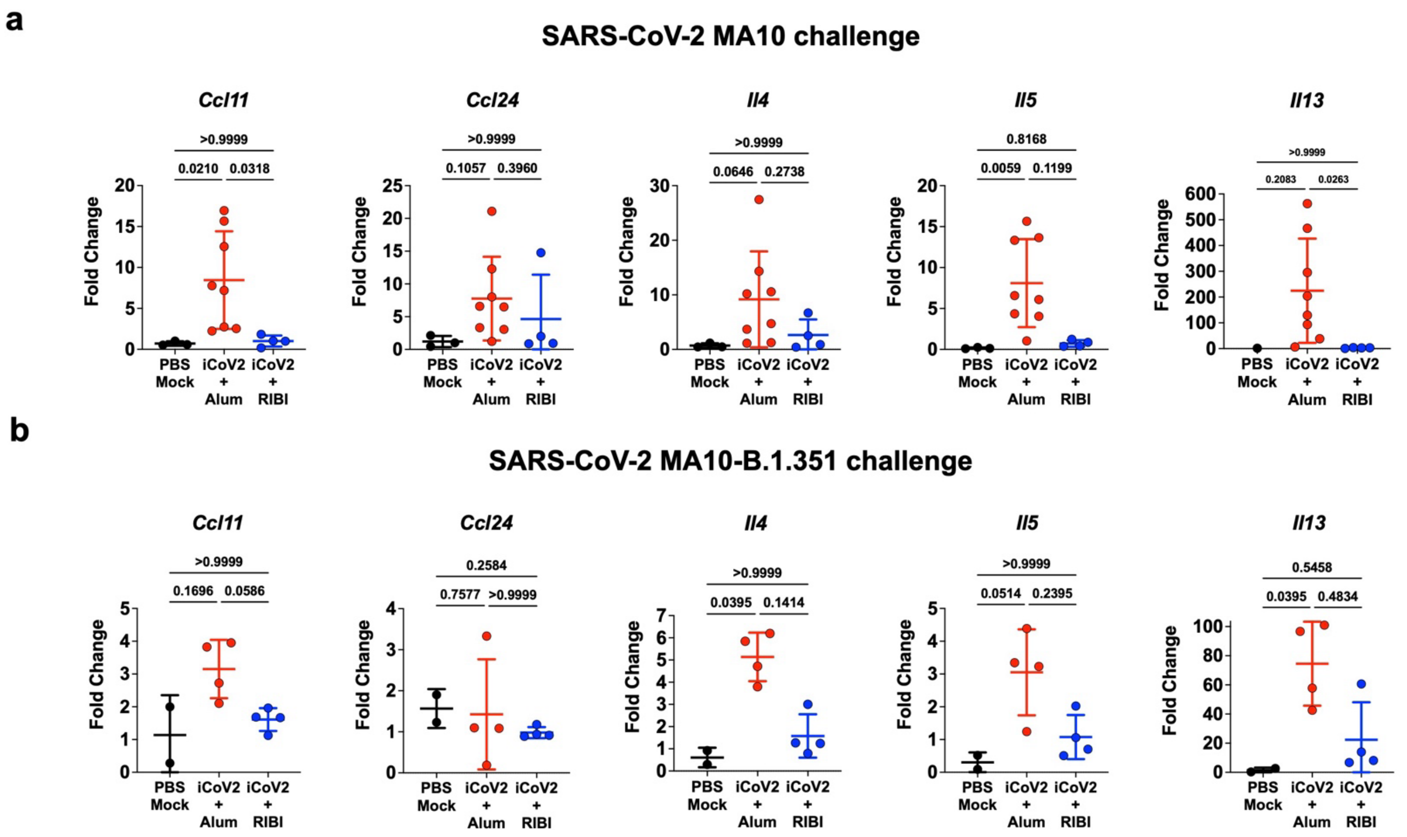

Supplementary Fig. 1: Type 2 cytokine gene expression during SARS-CoV-2 and B.1.351 infection.

Mice were mock-vaccinated with PBS or vaccinated with inactivated SARS-CoV-2 formulated with either Alum or RIBI adjuvants and challenged 3 weeks post-boost with either SARS-CoV-2 MA10 or SARS-CoV-2 MA10-B.1.351. Pulmonary tissue specimens were collected at 5 DPI. Expression of type 2 cytokine genes in pulmonary tissue was measured by qRT-PCR. Results reported as fold change normalized to *Gapdh* expression using ΔΔCt

a SARS-CoV-2 MA10 challenge. **b** SARS-CoV-2 MA10-B.1.351 challenge.

Individual data points represent independent biological replicates. Measurements were taken from discrete samples. Analyzed by Kruskal-Wallis test with Dunn's multiple comparisons correction. Solid horizontal lines overlaying data represent group means. Error bars represent group SD. Solid horizontal lines above data represent pairwise comparisons with p-values. Results from 1 animal experiment.

Number of animals used: **(a)** *Ccl11, Ccl24, Il4, Il5* – iFLU + Alum (3), iCoV2 + Alum (8), iCoV2 + RIBI (4); *Il13* – iFLU + Alum (1), iCoV2 + Alum (8), iCoV2 + RIBI (4). **(b)** *Ccl11, Ccl24, Il4, Il5, Il13* iFLU + Alum (2), iCoV2 + Alum (4), iCoV2 + RIBI (4).

Alum = aluminum hydroxide adjuvant*; Ccl11* = C-C motif chemokine 11 gene*; Ccl24* = C-C motif chemokine 24 gene; DPI = days post-infection; iCoV2 = inactivated SARS-CoV-2 vaccine; ; *Il4* = Interleukin 4 gene; *Il5* = Interleukin 5 gene; *Il13* = Interleukin 13 gene; MA10 = mouse-adapted SARS-CoV-2; MA10-B.1.351 = MA10 expressing B.1.351 (Beta variant) spike protein; PBS = phosphate-buffered saline; RIBI = Sigma Adjuvant System adjuvant; SD = standard deviation

Source data are provided as a Source Data file.

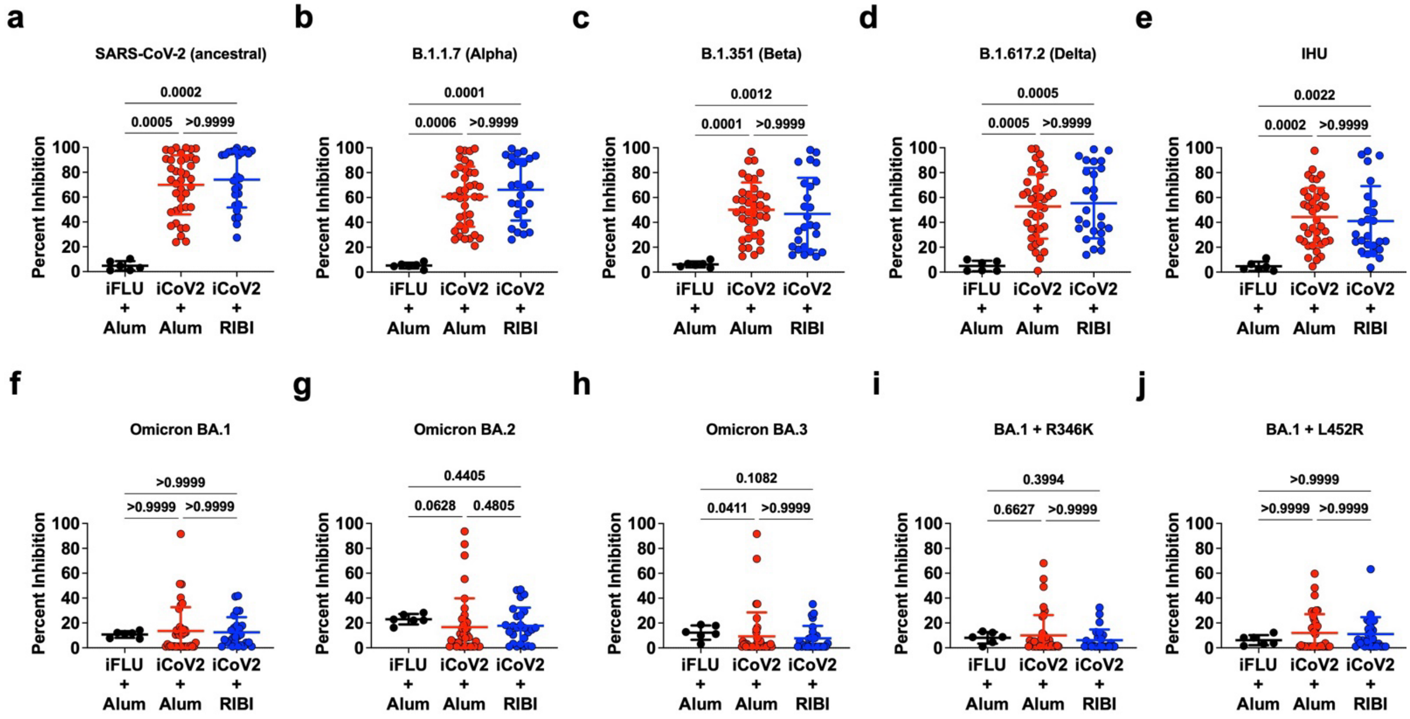

Supplementary Fig. 2: Vaccine immune serum surrogate neutralization of SARS-CoV-2 spike proteins.

Serum was collected from mice 3 weeks post-boost vaccination. Serum capacity for inhibition of ACE2 binding to SARS-CoV-2 and variant spike proteins was evaluated using the multiplexed Meso Scale Discovery (MSD) V-PLEX SARS-CoV-2 Panel 25 competition immunoassay as a surrogate neutralization assay.

a SARS-CoV-2 (ancestral); **b** SARS-CoV-2 B.1.1.7 (Alpha variant); **c** SARS-CoV-2 B.1.351 (Beta variant); **d** SARS-CoV-2 1.617.2 (Delta variant); **e** SARS-CoV-2 IHU variant; **f** SARS-CoV-2 Omicron BA.1; **g** SARS-CoV-2 BA.2; **h** SARS-CoV-2 BA.3; **i** SARS-CoV-2 BA.1 + R346K; **j** SARS-CoV-2 BA.1 + L452R.

Individual data points represent independent biological replicates. Measurements were taken from discrete samples. Combined results from 2 independent animal experiments (effects reproduced in each experiment). Analyzed by Kruskal-Wallis test with Dunn's multiple comparisons correction. Solid horizontal lines overlaying data represent group means. Error bars represent group SD. Dotted lines represent assay limit of detection. Solid horizontal lines above data represent pairwise comparisons with p-values.

Number of animals used: **(a-j)** iFLU + Alum (6), iCoV2 + Alum (39), iCoV2 + RIBI (26).

ACE2 = Angiotensin-converting enzyme 2; Alum = aluminum hydroxide adjuvant; iCoV2 = inactivated SARS-CoV-2 vaccine; iFLU = inactivated influenza virus vaccine; RIBI = Sigma Adjuvant System adjuvant; SD = standard deviation

Source data are provided as a Source Data file.

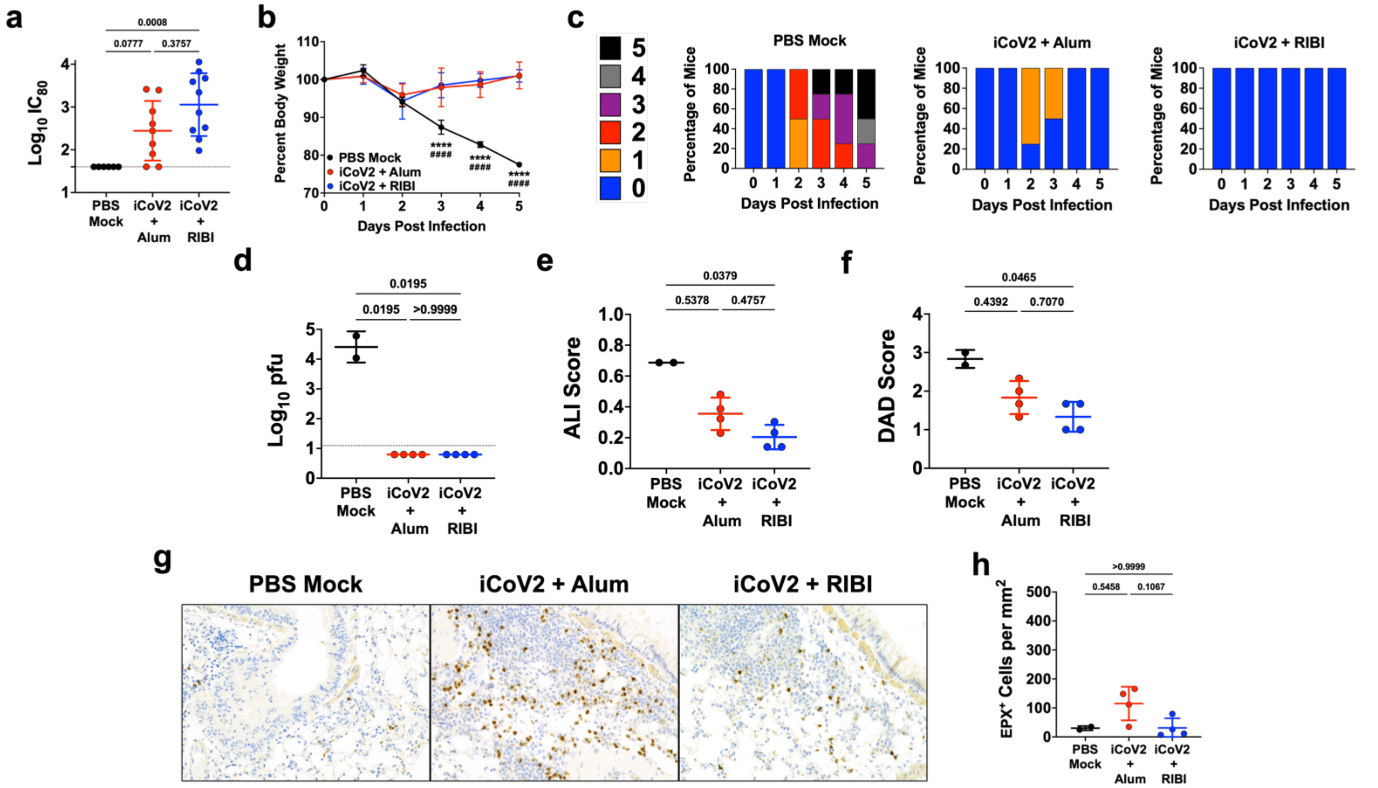

Supplementary Fig. 3: Safety and efficacy of inactivated SARS-CoV-2 vaccine against B.1.351.

Mice were either mock-vaccinated with PBS (PBS Mock) or vaccinated with inactivated SARS-CoV-2 plus aluminum hydroxide (Alum) or Sigma Adjuvant System adjuvant (RIBI) adjuvants and challenged 4 weeks post-boost with SARS-CoV-2 MA10-B.1.351. Pulmonary tissue was collected at 5 DPI.

a Post-boost serum samples were collected prior to challenge and neutralizing antibody titers against SARS-CoV-2 (B.1.351-S) were measured using a luminescence-based microneutralization assay; IC_{80} = 80% inhibitory concentration **b,c** Body weights (**b**) and clinical scores (**c**) were measured daily. **d** Pulmonary viral titers were quantified by plaque assay; pfu = plaque forming unit. **e, f** H&E-stained pulmonary specimens were analyzed to evaluate ALI (**e**) and DAD (**f**). **g, h** Representative photomicrographs (**g**) (magnification = 200X) and quantification (**h**) of pulmonary eosinophils immunohistochemically labeled for eosinophil peroxidase (EPX, brown cells).

a, d-f, h Individual data points represent independent biological replicates. Measurements were taken from discrete samples. Analyzed by Kruskal-Wallis test with Dunn's multiple comparisons correction. Solid horizontal lines and error bars overlaying data represent group means ± standard deviation (SD). Dotted lines in (**a**) and (**d**) represent assay limit of detection. Solid horizontal lines above data represent pairwise comparisons with p-values. Results from 1 (**b-h**) or 2 (**a**) independent animal experiments.

b, c Measurements were taken from individual animals repeatedly. Results in (**b**) reported as group mean ± SD. ****$p<0.0001$, PBS Mock versus iCoV2 + Alum, $^{\#\#\#\#}p<0.0001$, PBS Mock versus iCoV2 + RIBI, two-way ANOVA with Tukey's multiple comparisons correction.

Clinical scoring system: 0 = normal (blue); 1 = piloerection (orange); 2 = piloerection + kyphosis (red); 3 = piloerection, kyphosis and reduced movement (purple); 4 = markedly reduced movement and/or labored breathing (gray); 5 = moribund, dead or euthanized (black).

Number of animals used: **(a)** iFLU + Alum (6), iCoV2 + Alum (9), iCoV2 + RIBI (10). **(b, c)** iFLU + Alum (4), iCoV2 + Alum (4), iCoV2 + RIBI (4). **(d-h)** iFLU + Alum (2), iCoV2 + Alum (4), iCoV2 + RIBI (4).

Source data are provided as a Source Data file.

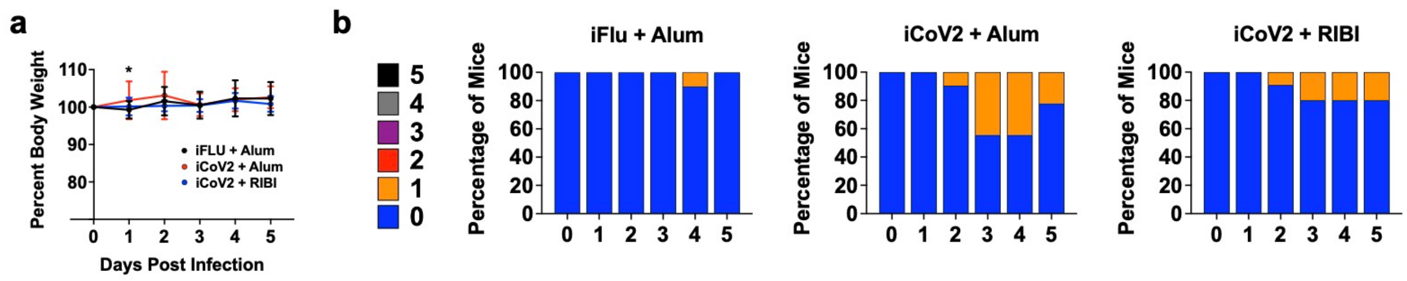

Supplementary Fig. 4: Clinical outcomes of Rs-SHC014-CoV challenge.

Vaccinated mice were challenged with Rs-SHC014-CoV at 4 weeks post-boost.

a Body weights were measured daily and reported as percent relative to baseline. **b** Clinical disease was evaluated daily using the clinical scoring system below. Measurements were taken from individual animals repeatedly. Combined results from 3 independent animal experiments (effects reproduced in each experiment). Results in (**a**) reported as group mean ± SD. *$p<0.05$, iFLU + Alum versus iCoV2 + Alum, two-way ANOVA with Tukey's multiple comparisons correction.

Number of animals used: **(a, b)** iFLU + Alum (22), iCoV2 + Alum (21), iCoV2 + RIBI (11).

Clinical scoring system: 0 = normal (blue), 1 = piloerection (orange), 2 = piloerection + kyphosis (red), 3 = piloerection, kyphosis and reduced movement (purple), 4 = markedly reduced movement and/or labored breathing (gray) and 5 = moribund, dead or euthanized (black).

Alum = aluminum hydroxide adjuvant; ANOVA = analysis of variance; DPI = days post-infection; iCoV2 = inactivated SARS-CoV-2 vaccine; iFLU = inactivated influenza virus vaccine; RIBI = Sigma Adjuvant System adjuvant; SD = standard deviation

Source data are provided as a Source Data file.

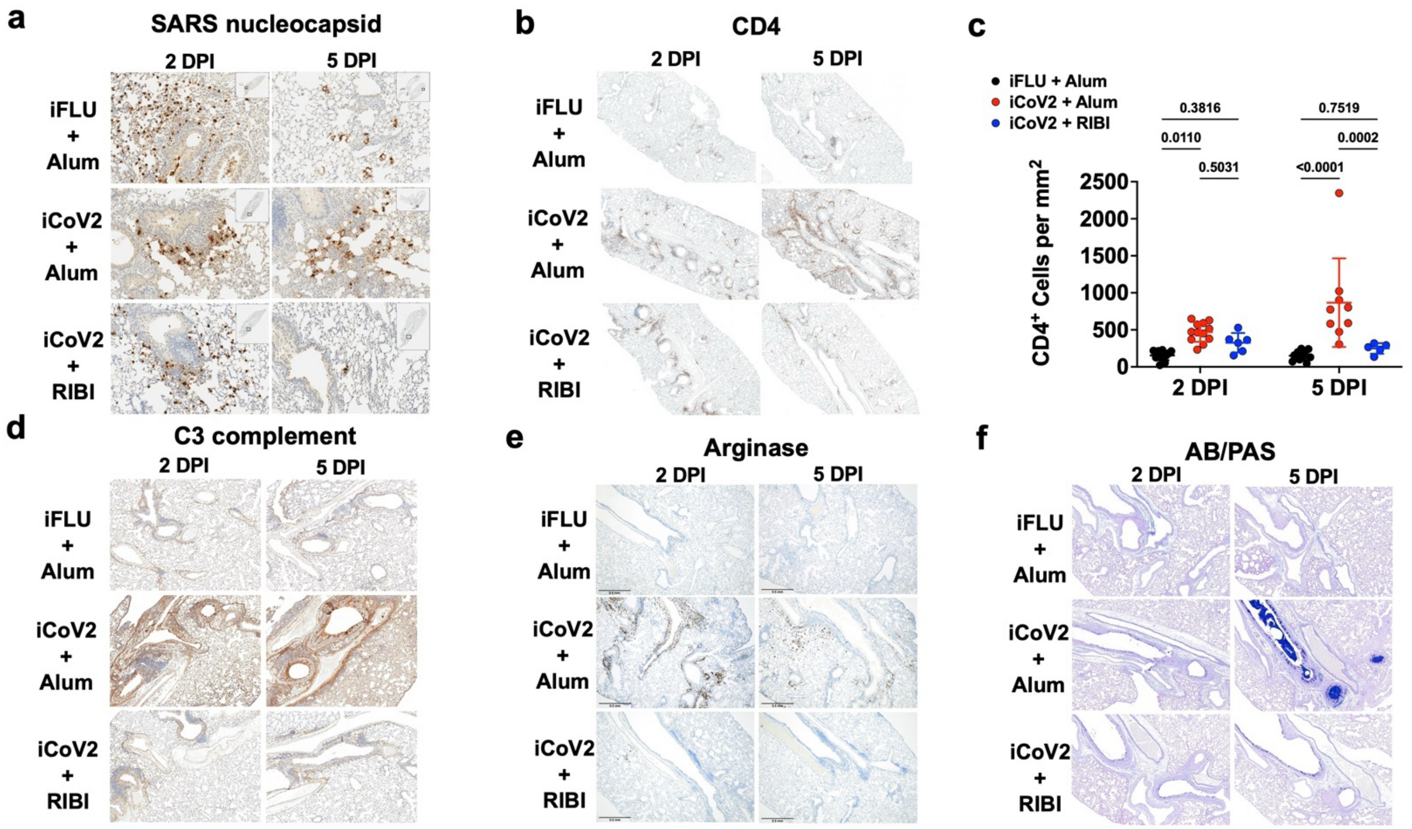

Supplementary Fig. 5: Viral load and inflammation during Rs-SHC014-CoV infection.

Vaccinated mice were challenged with Rs-SHC014-CoV at 4 weeks post-boost. Pulmonary tissue was collected at 2 and 5 DPI. Immunohistochemical staining was used to measure **a** SARS-CoV viral nucleocapsid antigen (100X magnification). **b** $CD4^+$ cells (20X magnification). **c** Quantitation of $CD4^+$ cells from (**b**). **d** C3 complement protein deposition (40X magnification). **e** $Arginase^+$ cells (40X magnification). **f** Alcian Blue Periodic Acid-Schiff staining was used to measure mucus (40X magnification).

a, b, d-f Micrographs from one experiment. Images acquired from discrete samples.

c Individual data points represent independent biological replicates. Measurements were taken from discrete samples. Analyzed by two-way ANOVA with Tukey's multiple comparisons correction. Solid horizontal lines overlaying data represent group means. Error bars represent group SD. Solid horizontal lines above data represent pairwise comparisons with p-values. Results from one animal experiment.

Number of animals used: **(a-e)** 2 DPI – iFLU + Alum (12), iCoV2 + Alum (12), iCoV2 + RIBI (6); 5 DPI – iFLU + Alum (12), iCoV2 + Alum (9), iCoV2 + RIBI (5). One representative micrograph chosen from each group for presentation.

AB/PAS = Alcian Blue - Periodic Acid Schiff; Alum = aluminum hydroxide adjuvant; ANOVA = analysis of variance; DPI = days post-infection; iCoV2 = inactivated SARS-CoV-2 vaccine; iFLU = inactivated influenza virus vaccine; RIBI = Sigma Adjuvant System adjuvant

Source data are provided as a Source Data file.

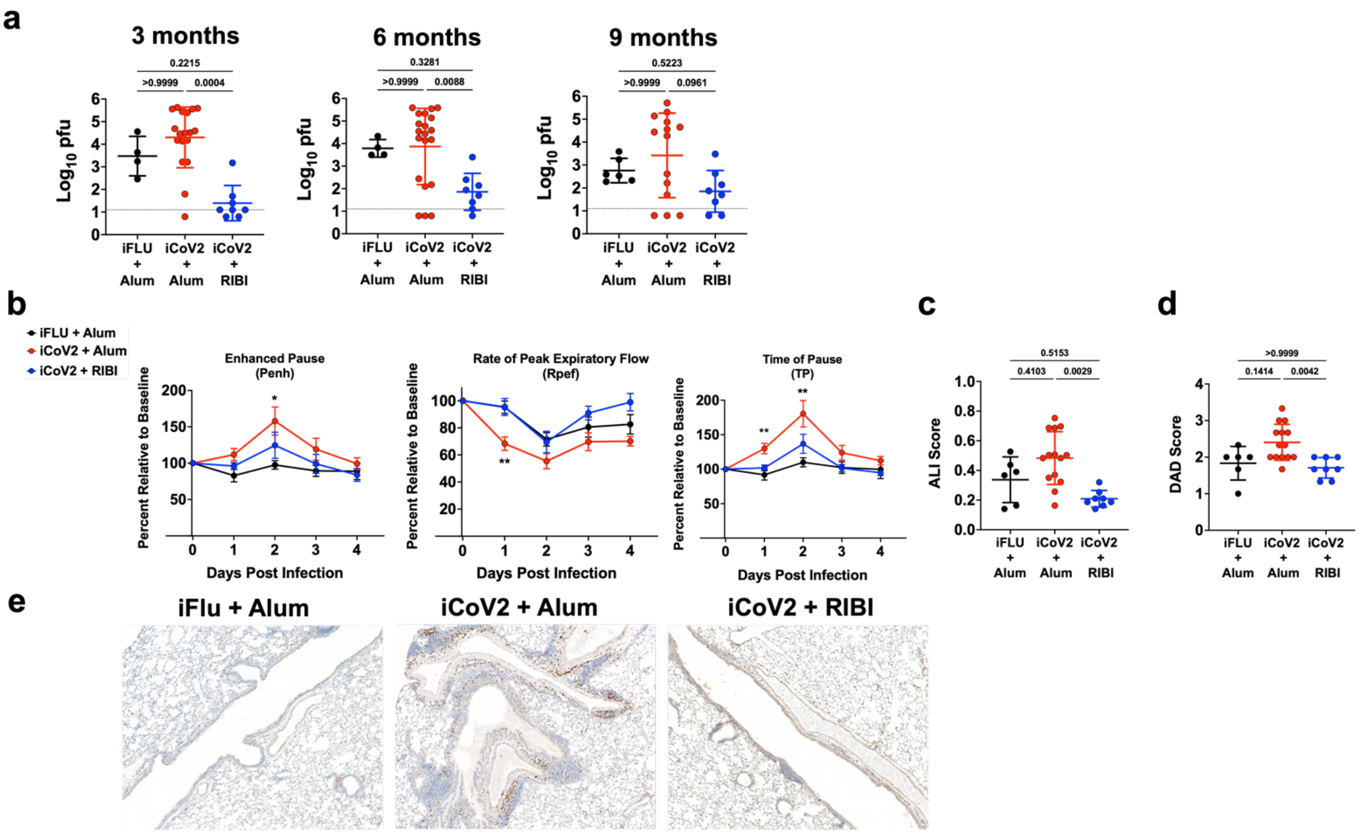

Supplementary Fig. 6: Long-term safety and efficacy of inactivated SARS-CoV-2 vaccine against Rs-SHC014-CoV.

Vaccinated mice were challenged with Rs-SHC014-CoV at 3-, 6-, 9- and 10.5-months post-boost. Pulmonary function was measured 1-4 DPI using whole body plethysmography and pulmonary tissue was collected from vaccinated mice at 5 DPI.

a Pulmonary viral titers shown for mice challenged at 3-, 6- and 9-months post-boost. Log-transformed results reported as $\log_{10}$ pfu. **b** Pulmonary function (**b**), acute lung injury (ALI) (**c**), and diffuse alveolar damage (DAD) (**d**) shown for mice challenged at 9 months post-boost. **e** Pulmonary eosinophils (eosinophil peroxidase $[EPX]^+$ cells) were measured at 10.5 months post-boost (latest collection time-point) using immunohistochemical staining (representative micrographs, 40X magnification; quantified in Fig. 2h).

a, c, d Individual data points represent independent biological replicates. Measurements were taken from discrete samples. Analyzed by Kruskal-Wallis test with Dunn's multiple comparisons correction. Solid horizontal lines overlaying data represent group mean group ± standard deviation. Dotted lines in (**a**) represent assay LOD. Solid horizontal lines above data represent pairwise comparisons with p-values. Results from multiple time points of one animal experiment.

b Measurements were taken from individual animals repeatedly. Reported as group mean ± SEM. *p<0.05, **p<0.01, iFLU + Alum versus iCoV2 + Alum, two-way ANOVA with Geisser-Greenhouse correction and Tukey's multiple comparisons correction. Results from one experiment.

Number of animals used: **(a)** 3 months – iFLU + Alum (4), iCoV2 + Alum (18), iCoV2 + RIBI (8); 6 months – iFLU + Alum (4), iCoV2 + Alum (20), iCoV2 + RIBI (8); 9 months – iFLU + Alum (6), iCoV2 + Alum (14), iCoV2 + RIBI (8). **(b-d)** iFLU + Alum (6), iCoV2 + Alum (14), iCoV2 + RIBI (8). **(e)** iFLU + Alum (5), iCoV2 + Alum (14), iCoV2 + RIBI (10).

Source data are provided as a Source Data file.

a

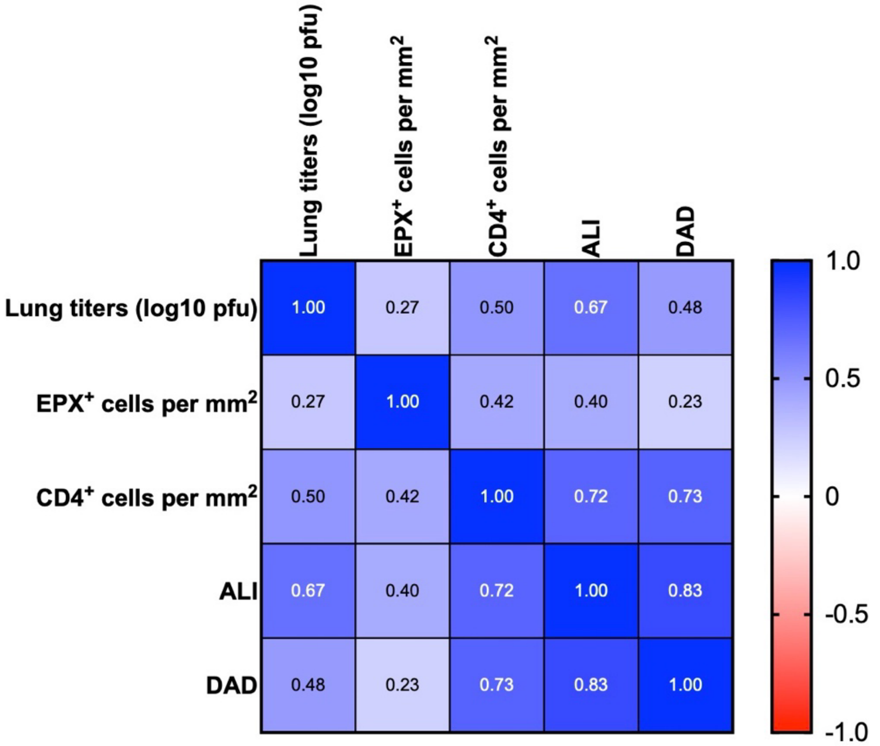

b

	Lung titers (log10 pfu)	EPX^+ cells per mm^2	$CD4^+$ cells per mm^2	ALI	DAD
Lung titers (log10 pfu)		0.3548	0.0708	0.0111	0.0852
EPX^+ cells per mm^2	0.3548		0.1413	0.1535	0.4235
$CD4^+$ cells per mm^2	0.0708	0.1413		0.0047	0.0038
ALI	0.0111	0.1535	0.0047		0.0004
DAD	0.0852	0.4235	0.0038	0.0004	

Supplementary Fig. 7: Correlation of vaccine-enhanced disease parameters during Rs-SHC014-CoV infection.

Mice vaccinated with iCoV2 + Alum were challenged with Rs-SHC014-CoV at 10.5-months post-boost. Pulmonary tissue was collected from vaccinated mice at 5 DPI. Pulmonary viral titers, eosinophils (eosinophil peroxidase $[EPX]^+$ cells), $CD4^+$ cells, ALI, and DAD were evaluated. Multivariable correlation analysis (Spearman) was performed to evaluate for correlation between various disease parameters.

a Multivariable correlation matrix with pairwise Spearman *r* values. **b** Pairwise multiplicity-adjusted p-values (two-tailed).

Number of animals used: (**a, b**) 14.

ALI = acute lung injury; Alum = aluminum hydroxide adjuvant; DAD = diffuse alveolar damage; DPI = days post-infection; EPX = eosinophil peroxidase; iCoV2 = inactivated SARS-CoV-2 vaccine; pfu = plaque-forming units

Source data are provided as a Source Data file.

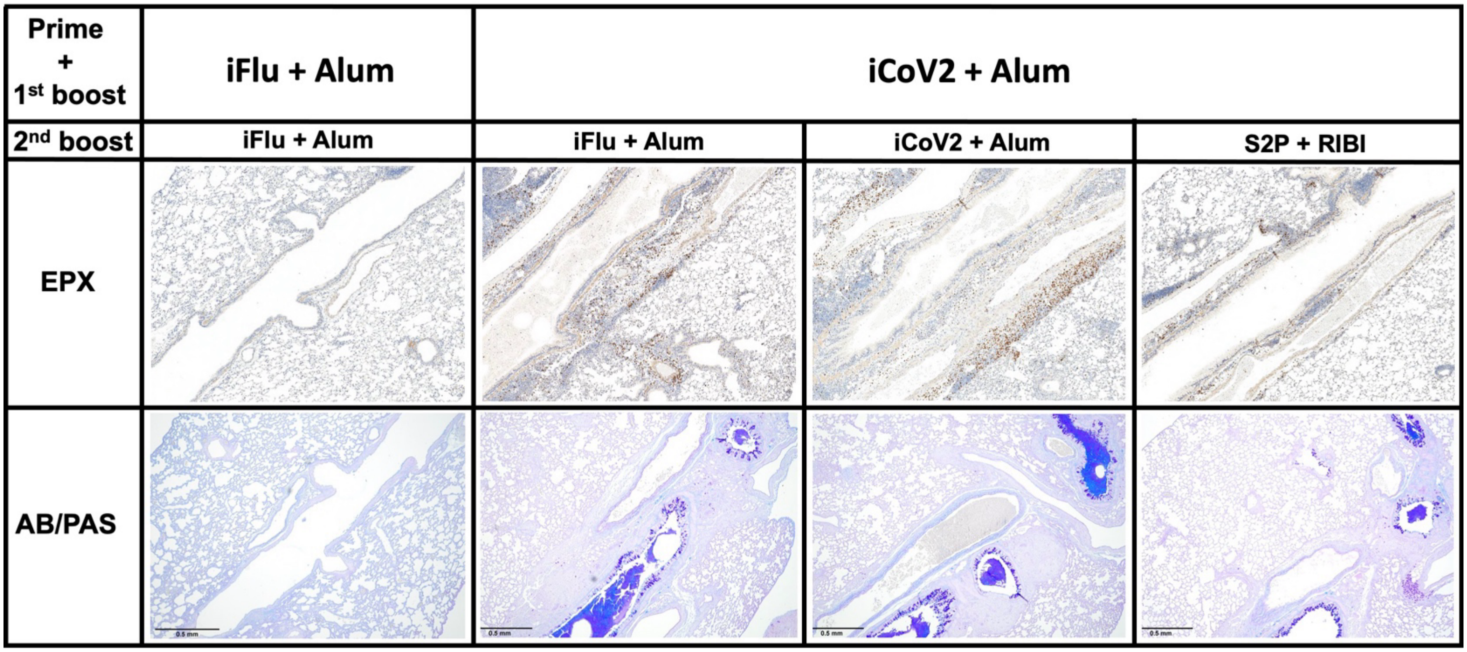

Supplementary Fig. 8: Type 2 inflammation during Rs-SHC014-CoV infection following alternative boost vaccination.

Approximately 9.5 months post-boost vaccination, mice received a third dose of iCoV2 + Alum or were boosted with a heterologous vaccine formulation (S2P + RIBI). Four weeks post-second boost, mice were challenged with Rs-SHC014-CoV. Pulmonary tissue was collected 5 DPI.

Eosinophils (eosinophil peroxidase $[EPX]^+$ cells) were measured using immunohistochemical staining. Mucus was measured using Alcian Blue Periodic Acid-Schiff staining. Representative micrographs (scale bar = 0.5mm; EPX quantified in Fig. 4e) from 1 experiment. Images acquired from discrete samples.

Number of animals used: iFLU + Alum, iFLU + Alum (5), iCoV2 + Alum, iFLU + Alum (14), iCoV2 + Alum, iCoV2 + Alum (10), iCoV2 + Alum, S2P + RIBI (10). One representative micrograph chosen from each group for presentation.

AB/PAS = Alcian Blue - Periodic Acid Schiff; Alum = aluminum hydroxide adjuvant; DPI = days post-infection; EPX = eosinophil peroxidase; iCoV2 = inactivated SARS-CoV-2 vaccine; iFLU = inactivated influenza virus vaccine; RIBI = Sigma Adjuvant System adjuvant; S2P = pre-fusion stabilized SARS-CoV-2 spike protein

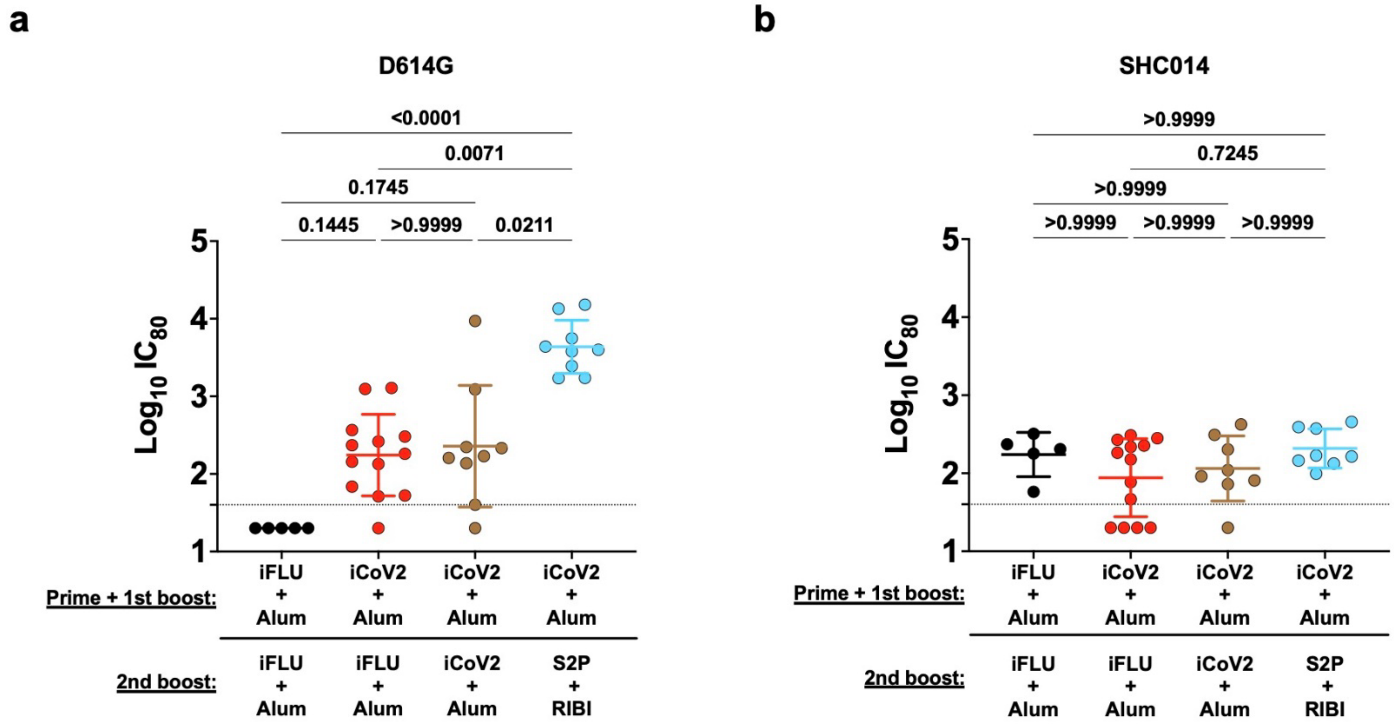

Supplementary Fig. 9: Serum neutralization titers induced by alternative boost vaccines.

Mice were initially vaccinated with inactivated SARS-CoV-2 and aluminum hydroxide adjuvant (iCoV2 + Alum). Approximately 9.5 months post-boost vaccination, mice were administered either a third dose of iCoV2 + Alum (homologous second boost) or were boosted with an alternative vaccine formulation (recombinant full-length pre-fusion stabilized spike protein [S2P] + RIBI) 4 weeks prior to challenge with Rs-SHC014-CoV (SHC014). Post-boost serum samples were collected prior to challenge and neutralizing antibody titers against SARS-CoV-2 (D614G) (**a**) or SHC014 (**b**) were measured using a luminescence-based microneutralization assay.

a, b Log-transformed serum neutralization titers reported as IC_{80}. Individual data points represent independent biological replicates. Measurements were taken from discrete samples. Results from 1 animal experiment. Analyzed by Kruskal-Wallis test with Dunn's multiple comparisons correction. Solid horizontal lines overlaying data represent group means. Error bars represent group SD. Dotted lines represent assay LOD. Solid horizontal lines above data represent pairwise comparisons with p-values.

Number of animals used: **(a)** iFLU + Alum, iFLU + Alum (5); iCoV2 + Alum, iFLU + Alum (13), iCoV2 + Alum, iCoV2 + Alum (9); iCoV2 + Alum, S2P + RIBI (9). **(b)** iFLU + Alum, iFLU + Alum (5), iCoV2 + Alum, iFLU + Alum (13), iCoV2 + Alum, iCoV2 + Alum (8), iCoV2 + Alum, S2P + RIBI (8).

Alum = aluminum hydroxide adjuvant; IC_{80} = 80% serum reciprocal inhibitory dilution titer; iCoV2 = inactivated SARS-CoV-2 vaccine; iFLU = inactivated influenza virus vaccine; LOD = limit of detection; RIBI = Sigma Adjuvant System adjuvant; S2P = pre-fusion stabilized SARS-CoV-2 spike protein; SD = standard deviation;

Source data are provided as a Source Data file.

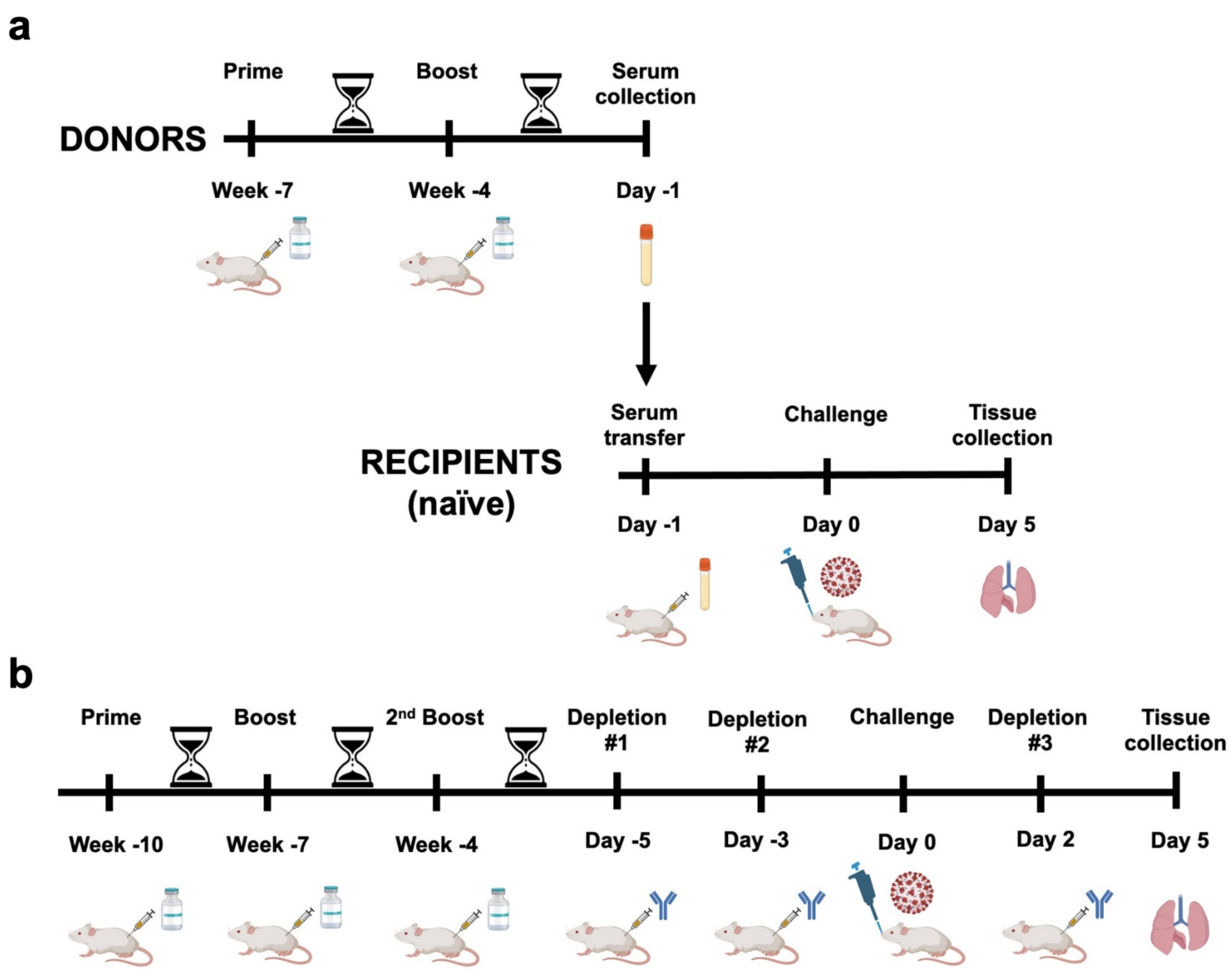

Supplementary Fig. 10: Experimental outlines for passive serum transfer and $CD4^+$ cell depletion.

a Donor mice were vaccinated with iCoV2 (+ Alum or RIBI). Four weeks post-boost, serum was collected from donor mice and transferred to naïve recipient mice 1 day prior to challenge with Rs-SHC014-CoV. Pulmonary function was measured 1-4 DPI using WBP and pulmonary tissue was collected at 5 DPI.

b Mice were vaccinated using the double-boost method with iCoV2 + Alum. To deplete $CD4^+$ T_H cells, anti-CD4 monoclonal antibody (GK1.5) was administered to mice before and during viral challenge with Rs-SHC014-CoV. Pulmonary function was measured 1-4 DPI using WBP and pulmonary tissue was collected at 5 DPI.

Alum = aluminum hydroxide adjuvant; DPI = days post-infection; iCoV2 = inactivated SARS-CoV-2 vaccine; T_H = T helper; WBP = Whole body plethysmography

Created with Biorender.

Supplementary Table 1: Pulmonary gene pathway enrichment during Rs-SHC014-CoV infection.

Vaccinated mice were challenged with Rs-SHC014-CoV 4 weeks post-boost. Pulmonary tissue was collected from vaccinated mice at 2 or 5 DPI. RNA-Seq and Gene Ontology Enrichment analysis were performed using whole pulmonary tissue. Pathways displayed are upregulated or downregulated (+/-) in iCoV2 + Alum- or iCoV2 + RIBI-vaccinated mice compared to mice in the opposite vaccine group (e.g., 5DPI_iCoV2 + Alum = upregulated at 5 DPI in iCoV2 + Alum-vaccinated mice compared to iCoV2 + RIBI-vaccinated mice). Analysis was performed on discrete samples. Data analysis is described in detail in RNA Sequencing (RNA-Seq) in Methods.

Number of animals used: 2 DPI – iCoV2 + Alum (12), iCoV2 + RIBI (6); 5 DPI – iCoV2 + Alum (9), iCoV2 + RIBI (5).

Alum = aluminum hydroxide adjuvant; DPI = days post-infection; FDR = False Discovery Rate; GO = Gene Ontology; iCoV2 = inactivated SARS-CoV-2 vaccine; RIBI = Sigma Adjuvant System adjuvant

GO Term Name	GO ID	REF Gene List	Observed Genes	Expected Genes	+/-	Fold Enrichment	Enrichment p-value	Enrichment FDR	DPI & Vaccine
**complement component C3b binding**	GO:0001851	9	1	0	+	> 100	5.44E-03	1E+00	2DPiCoV2 + RIBI
**carbohydrate binding**	GO:0030246	283	2	0.15	+	12.95	1.01E-02	1E+00	2DPI_iCoV2 + RIBI
**polypeptide N-acetylgalactosaminyltransferase activity**	GO:0004653	18	1	0.01	+	> 100	1.03E-02	1E+00	2DPI_iCoV2 + RIBI
**opsonin binding**	GO:0001846	22	1	0.01	+	83.32	1.25E-02	1E+00	2DPI_iCoV2 + RIBI
**complement binding**	GO:0001848	28	1	0.02	+	65.47	1.57E-02	1E+00	2DPI_iCoV2 + RIBI
**polysaccharide binding**	GO:0030247	28	1	0.02	+	65.47	1.57E-02	1E+00	2DPI_iCoV2 + RIBI
**acetylgalactosaminyltransferase activity**	GO:0008376	33	1	0.02	+	55.55	1.84E-02	1E+00	2DPI_iCoV2 + RIBI
**carbohydrate transmembrane transporter activity**	GO:0015144	38	1	0.02	+	48.24	2.11E-02	1E+00	2DPI_iCoV2 + RIBI
**calcium channel regulator activity**	GO:0005246	42	1	0.02	+	43.64	2.32E-02	1E+00	2DPI_iCoV2 + RIBI
**scavenger receptor activity**	GO:0005044	43	1	0.02	+	42.63	2.37E-02	1E+00	2DPI_iCoV2 + RIBI
**E-box binding**	GO:0070888	58	1	0.03	+	31.6	3.17E-02	1E+00	2DPI_iCoV2 + RIBI
**cargo receptor activity**	GO:0038024	82	1	0.04	+	22.35	4.43E-02	1E+00	2DPI_iCoV2 + RIBI
**cytokine activity**	GO:0005125	231	11	0.8	+	13.78	6.7E-10	3.35E-06	2DPI_iCoV2 + Alum
**receptor ligand activity**	GO:0048018	534	14	1.84	+	7.59	4.8E-09	1.2E-05	2DPI_iCoV2 + Alum
**signaling receptor activator activity**	GO:0030546	545	14	1.88	+	7.44	6.17E-09	1.03E-05	2DPI_iCoV2 + Alum
**chemokine activity**	GO:0008009	42	6	0.15	+	41.35	1.49E-08	1.86E-05	2DPI_iCoV2 + Alum
**signaling receptor regulator activity**	GO:0030545	591	14	2.04	+	6.86	1.67E-08	1.67E-05	2DPI_iCoV2 + Alum
**CCR chemokine receptor binding**	GO:0048020	58	6	0.2	+	29.94	8.72E-08	7.25E-05	2DPI_iCoV2 + Alum
**cytokine receptor binding**	GO:0005126	316	10	1.09	+	9.16	1.73E-07	1.24E-04	2DPI_iCoV2 + Alum

**chemokine receptor binding**	GO:0042379	79	6	0.27	+	21.98	4.8E-07	3E-04	2DPI_iCoV2 + Alum
**molecular function regulator activity**	GO:0098772	2147	23	7.42	+	3.1	5.9E-07	3.27E-04	2DPI_iCoV2 + Alum
**signaling receptor binding**	GO:0005102	1650	19	5.7	+	3.33	2.62E-06	1.31E-03	2DPI_iCoV2 + Alum
**molecular function activator activity**	GO:0140677	1192	16	4.12	+	3.89	2.83E-06	1.28E-03	2DPI_iCoV2 + Alum
**chitin binding**	GO:0008061	11	3	0.04	+	78.94	1.39E-05	5.78E-03	2DPI_iCoV2 + Alum
**G protein-coupled receptor binding**	GO:0001664	328	8	1.13	+	7.06	2.03E-05	7.81E-03	2DPI_iCoV2 + Alum
**carbohydrate binding**	GO:0030246	283	7	0.98	+	7.16	6.33E-05	2.26E-02	2DPI_iCoV2 + Alum
**CCR3 chemokine receptor binding**	GO:0031728	5	2	0.02	+	> 100	2.43E-04	8.08E-02	2DPI_iCoV2 + Alum
**brain-derived neurotrophic factor binding**	GO:0048403	5	2	0.02	+	> 100	2.43E-04	7.58E-02	2DPI_iCoV2 + Alum
**mannose binding**	GO:0005537	33	3	0.11	+	26.31	2.58E-04	7.58E-02	2DPI_iCoV2 + Alum
**protein binding**	GO:0005515	9462	48	32.69	+	1.47	4.56E-04	1.27E-01	2DPI_iCoV2 + Alum
**serine-type endopeptidase activity**	GO:0004252	194	5	0.67	+	7.46	6.23E-04	1.64E-01	2DPI_iCoV2 + Alum
**nerve growth factor binding**	GO:0048406	9	2	0.03	+	64.32	6.31E-04	1.57E-01	2DPI_iCoV2 + Alum
**serine-type peptidase activity**	GO:0008236	211	5	0.73	+	6.86	9.01E-04	2.14E-01	2DPI_iCoV2 + Alum
**serine hydrolase activity**	GO:0017171	216	5	0.75	+	6.7	9.98E-04	2.26E-01	2DPI_iCoV2 + Alum
**serine-type endopeptidase inhibitor activity**	GO:0004867	129	4	0.45	+	8.97	1.16E-03	2.51E-01	2DPI_iCoV2 + Alum
**neurotrophin binding**	GO:0043121	13	2	0.04	+	44.53	1.19E-03	2.48E-01	2DPI_iCoV2 + Alum
**growth factor activity**	GO:0008083	149	4	0.51	+	7.77	1.93E-03	3.86E-01	2DPI_iCoV2 + Alum
**adrenergic receptor binding**	GO:0031690	23	2	0.08	+	25.17	3.33E-03	6.4E-01	2DPI_iCoV2 + Alum
**oxidoreductase activity, acting on single donors with incorporation of molecular oxygen, incorporation of two atoms of oxygen**	GO:0016702	26	2	0.09	+	22.26	4.17E-03	7.71E-01	2DPI_iCoV2 + Alum
**monosaccharide binding**	GO:0048029	92	3	0.32	+	9.44	4.33E-03	7.71E-01	2DPI_iCoV2 + Alum
**oxidoreductase activity, acting on single donors with incorporation of molecular oxygen**	GO:0016701	27	2	0.09	+	21.44	4.47E-03	7.7E-01	2DPI_iCoV2 + Alum
**endopeptidase inhibitor activity**	GO:0004866	195	4	0.67	+	5.94	4.96E-03	8.25E-01	2DPI_iCoV2 + Alum
**endopeptidase activity**	GO:0004175	453	6	1.57	+	3.83	5.02E-03	8.08E-01	2DPI_iCoV2 + Alum
**peptidase inhibitor activity**	GO:0030414	202	4	0.7	+	5.73	5.6E-03	8.73E-01	2DPI_iCoV2 + Alum
**endopeptidase regulator activity**	GO:0061135	214	4	0.74	+	5.41	6.82E-03	1E+00	2DPI_iCoV2 + Alum
**chemokine binding**	GO:0019956	34	2	0.12	+	17.03	6.83E-03	1E+00	2DPI_iCoV2 + Alum
**galactose:sodium symporter activity**	GO:0015371	1	1	0	+	> 100	6.87E-03	9.8E-01	2DPI_iCoV2 + Alum

**CCR4 chemokine receptor binding**	GO:0031729	1	1	0	+	> 100	6.87E-03	9.53E-01	2DPI_iCoV2 + Alum
**glycine amidinotransferase activity**	GO:0015068	1	1	0	+	> 100	6.87E-03	9.27E-01	2DPI_iCoV2 + Alum
**amidinotransferase activity**	GO:0015067	1	1	0	+	> 100	6.87E-03	9.03E-01	2DPI_iCoV2 + Alum
**interleukin-13 receptor binding**	GO:0005144	1	1	0	+	> 100	6.87E-03	8.8E-01	2DPI_iCoV2 + Alum
**peptidase activity**	GO:0008233	642	7	2.22	+	3.16	6.97E-03	8.7E-01	2DPI_iCoV2 + Alum
**virus receptor activity**	GO:0001618	41	2	0.14	+	14.12	9.64E-03	1E+00	2DPI_iCoV2 + Alum
**exogenous protein binding**	GO:0140272	42	2	0.15	+	13.78	1.01E-02	1E+00	2DPI_iCoV2 + Alum
**inorganic diphosphate transmembrane transporter activity**	GO:0030504	2	1	0.01	+	> 100	1.03E-02	1E+00	2DPI_iCoV2 + Alum
**arginase activity**	GO:0004053	2	1	0.01	+	> 100	1.03E-02	1E+00	2DPI_iCoV2 + Alum
**netrin receptor binding**	GO:1990890	2	1	0.01	+	> 100	1.03E-02	1E+00	2DPI_iCoV2 + Alum
**alpha-2C adrenergic receptor binding**	GO:0031696	2	1	0.01	+	> 100	1.03E-02	1E+00	2DPI_iCoV2 + Alum
**alpha-glucoside transmembrane transporter activity**	GO:0015151	2	1	0.01	+	> 100	1.03E-02	1E+00	2DPI_iCoV2 + Alum
**interleukin-5 receptor binding**	GO:0005137	2	1	0.01	+	> 100	1.03E-02	1E+00	2DPI_iCoV2 + Alum
**interleukin-8 binding**	GO:0019959	3	1	0.01	+	96.48	1.37E-02	1E+00	2DPI_iCoV2 + Alum
**alpha2-adrenergic receptor activity**	GO:0004938	3	1	0.01	+	96.48	1.37E-02	1E+00	2DPI_iCoV2 + Alum
**iodide transmembrane transporter activity**	GO:0015111	3	1	0.01	+	96.48	1.37E-02	1E+00	2DPI_iCoV2 + Alum
**tryptophan 2,3-dioxygenase activity**	GO:0004833	3	1	0.01	+	96.48	1.37E-02	1E+00	2DPI_iCoV2 + Alum
**glucoside transmembrane transporter activity**	GO:0042947	3	1	0.01	+	96.48	1.37E-02	1E+00	2DPI_iCoV2 + Alum
**interleukin-4 receptor binding**	GO:0005136	3	1	0.01	+	96.48	1.37E-02	1E+00	2DPI_iCoV2 + Alum
**cytokine binding**	GO:0019955	142	3	0.49	+	6.11	1.38E-02	1E+00	2DPI_iCoV2 + Alum
**peptidase regulator activity**	GO:0061134	267	4	0.92	+	4.34	1.43E-02	1E+00	2DPI_iCoV2 + Alum
**sulfur compound transmembrane transporter activity**	GO:1901682	54	2	0.19	+	10.72	1.6E-02	1E+00	2DPI_iCoV2 + Alum
**growth factor receptor binding**	GO:0070851	151	3	0.52	+	5.75	1.62E-02	1E+00	2DPI_iCoV2 + Alum
**channel regulator activity**	GO:0016247	154	3	0.53	+	5.64	1.7E-02	1E+00	2DPI_iCoV2 + Alum
**arachidonate 15-lipoxygenase activity**	GO:0050473	4	1	0.01	+	72.36	1.71E-02	1E+00	2DPI_iCoV2 + Alum
**N-acetyl-beta-D-galactosaminidase activity**	GO:0102148	4	1	0.01	+	72.36	1.71E-02	1E+00	2DPI_iCoV2 + Alum
**group III metabotropic glutamate receptor activity**	GO:0001642	4	1	0.01	+	72.36	1.71E-02	1E+00	2DPI_iCoV2 + Alum
**linoleate 13S-lipoxygenase activity**	GO:0016165	4	1	0.01	+	72.36	1.71E-02	1E+00	2DPI_iCoV2 + Alum

**arachidonate 12-lipoxygenase activity**	GO:0004052	4	1	0.01	+	72.36	1.71E-02	1E+00	2DPI_iCoV2 + Alum
**histone acetyltransferase regulator activity**	GO:0035034	4	1	0.01	+	72.36	1.71E-02	1E+00	2DPI_iCoV2 + Alum
**norepinephrine binding**	GO:0051380	4	1	0.01	+	72.36	1.71E-02	1E+00	2DPI_iCoV2 + Alum
**alpha-2A adrenergic receptor binding**	GO:0031694	4	1	0.01	+	72.36	1.71E-02	1E+00	2DPI_iCoV2 + Alum
**extracellular matrix binding**	GO:0050840	57	2	0.2	+	10.16	1.76E-02	1E+00	2DPI_iCoV2 + Alum
**tumor necrosis factor binding**	GO:0043120	5	1	0.02	+	57.89	2.05E-02	1E+00	2DPI_iCoV2 + Alum
**guanylate cyclase activator activity**	GO:0030250	5	1	0.02	+	57.89	2.05E-02	1E+00	2DPI_iCoV2 + Alum
**galactose transmembrane transporter activity**	GO:0005354	5	1	0.02	+	57.89	2.05E-02	1E+00	2DPI_iCoV2 + Alum
**type 1 fibroblast growth factor receptor binding**	GO:0005105	6	1	0.02	+	48.24	2.39E-02	1E+00	2DPI_iCoV2 + Alum
**chitinase activity**	GO:0004568	6	1	0.02	+	48.24	2.39E-02	1E+00	2DPI_iCoV2 + Alum
**beta-N-acetylhexosaminidase activity**	GO:0004563	6	1	0.02	+	48.24	2.39E-02	1E+00	2DPI_iCoV2 + Alum
**guanylate cyclase regulator activity**	GO:0030249	6	1	0.02	+	48.24	2.39E-02	1E+00	2DPI_iCoV2 + Alum
**epinephrine binding**	GO:0051379	6	1	0.02	+	48.24	2.39E-02	1E+00	2DPI_iCoV2 + Alum
**alpha-adrenergic receptor activity**	GO:0004936	6	1	0.02	+	48.24	2.39E-02	1E+00	2DPI_iCoV2 + Alum
**fucose binding**	GO:0042806	7	1	0.02	+	41.35	2.72E-02	1E+00	2DPI_iCoV2 + Alum
**heterotrimeric G-protein binding**	GO:0032795	7	1	0.02	+	41.35	2.72E-02	1E+00	2DPI_iCoV2 + Alum
**interleukin-1 binding**	GO:0019966	7	1	0.02	+	41.35	2.72E-02	1E+00	2DPI_iCoV2 + Alum
**C-X-C chemokine binding**	GO:0019958	7	1	0.02	+	41.35	2.72E-02	1E+00	2DPI_iCoV2 + Alum
**ABC-type glutathione S-conjugate transporter activity**	GO:0015431	7	1	0.02	+	41.35	2.72E-02	1E+00	2DPI_iCoV2 + Alum
**thyroid hormone binding**	GO:0070324	7	1	0.02	+	41.35	2.72E-02	1E+00	2DPI_iCoV2 + Alum
**binding**	GO:0005488	14105	58	48.73	+	1.19	3.04E-02	1E+00	2DPI_iCoV2 + Alum
**D-glucose transmembrane transporter activity**	GO:0055056	8	1	0.03	+	36.18	3.06E-02	1E+00	2DPI_iCoV2 + Alum
**RNA binding**	GO:0003723	1113	0	3.85	-	< 0.01	3.35E-02	1E+00	2DPI_iCoV2 + Alum
**adenylate cyclase inhibiting G protein-coupled glutamate receptor activity**	GO:0001640	9	1	0.03	+	32.16	3.39E-02	1E+00	2DPI_iCoV2 + Alum
**3-chloroallyl aldehyde dehydrogenase activity**	GO:0004028	9	1	0.03	+	32.16	3.39E-02	1E+00	2DPI_iCoV2 + Alum
**G protein-coupled glutamate receptor activity**	GO:0098988	9	1	0.03	+	32.16	3.39E-02	1E+00	2DPI_iCoV2 + Alum
**glucose:sodium symporter activity**	GO:0005412	9	1	0.03	+	32.16	3.39E-02	1E+00	2DPI_iCoV2 + Alum
**adrenergic receptor activity**	GO:0004935	9	1	0.03	+	32.16	3.39E-02	1E+00	2DPI_iCoV2 + Alum
**chloride:bicarbonate antiporter activity**	GO:0140900	9	1	0.03	+	32.16	3.39E-02	1E+00	2DPI_iCoV2 + Alum

**bicarbonate:monoatomic anion antiporter activity**	GO:0140829	9	1	0.03	+	32.16	3.39E-02	1E+00	2DPI_iCoV2 + Alum
**olfactory receptor activity**	GO:0004984	1169	0	4.04	-	< 0.01	3.46E-02	1E+00	2DPI_iCoV2 + Alum
**cyclase activator activity**	GO:0010853	10	1	0.03	+	28.94	3.72E-02	1E+00	2DPI_iCoV2 + Alum
**thioesterase binding**	GO:0031996	10	1	0.03	+	28.94	3.72E-02	1E+00	2DPI_iCoV2 + Alum
**secondary active sulfate transmembrane transporter activity**	GO:0008271	10	1	0.03	+	28.94	3.72E-02	1E+00	2DPI_iCoV2 + Alum
**oxalate transmembrane transporter activity**	GO:0019531	10	1	0.03	+	28.94	3.72E-02	1E+00	2DPI_iCoV2 + Alum
**hydrolase activity**	GO:0016787	2363	14	8.16	+	1.71	3.99E-02	1E+00	2DPI_iCoV2 + Alum
**hydrolase activity, acting on carbon-nitrogen bonds, in linear amidines**	GO:0016813	11	1	0.04	+	26.31	4.06E-02	1E+00	2DPI_iCoV2 + Alum
**dioxygenase activity**	GO:0051213	94	2	0.32	+	6.16	4.33E-02	1E+00	2DPI_iCoV2 + Alum
**acetylcholine binding**	GO:0042166	12	1	0.04	+	24.12	4.39E-02	1E+00	2DPI_iCoV2 + Alum
**omega peptidase activity**	GO:0008242	12	1	0.04	+	24.12	4.39E-02	1E+00	2DPI_iCoV2 + Alum
**cytokine receptor activity**	GO:0004896	95	2	0.33	+	6.09	4.42E-02	1E+00	2DPI_iCoV2 + Alum
**chloride channel regulator activity**	GO:0017081	13	1	0.04	+	22.26	4.72E-02	1E+00	2DPI_iCoV2 + Alum
**catecholamine binding**	GO:1901338	13	1	0.04	+	22.26	4.72E-02	1E+00	2DPI_iCoV2 + Alum
**hydrolase activity, hydrolyzing O-glycosyl compounds**	GO:0004553	100	2	0.35	+	5.79	4.83E-02	1E+00	2DPI_iCoV2 + Alum
**ATPase binding**	GO:0051117	100	2	0.35	+	5.79	4.83E-02	1E+00	2DPI_iCoV2 + Alum
**olfactory receptor activity**	GO:0004984	1169	0	15.04	-	< 0.01	5.17E-07	2.58E-03	5DPI_iCoV2 + RIBI
**structural constituent of muscle**	GO:0008307	21	6	0.27	+	22.21	9.42E-07	2.35E-03	5DPI_iCoV2 + RIBI
**actinin binding**	GO:0042805	41	7	0.53	+	13.27	2.34E-06	3.89E-03	5DPI_iCoV2 + RIBI
**actin binding**	GO:0003779	448	20	5.76	+	3.47	2.6E-06	3.24E-03	5DPI_iCoV2 + RIBI
**alpha-actinin binding**	GO:0051393	30	6	0.39	+	15.55	5.63E-06	5.62E-03	5DPI_iCoV2 + RIBI
**cytoskeletal protein binding**	GO:0008092	1021	31	13.14	+	2.36	1.35E-05	1.12E-02	5DPI_iCoV2 + RIBI
**carboxylic acid binding**	GO:0031406	217	12	2.79	+	4.3	3.93E-05	2.8E-02	5DPI_iCoV2 + RIBI
**titin binding**	GO:0031432	12	4	0.15	+	25.91	4.11E-05	2.57E-02	5DPI_iCoV2 + RIBI
**binding**	GO:0005488	14105	214	181.47	+	1.18	4.59E-05	2.55E-02	5DPI_iCoV2 + RIBI
**potassium channel activity**	GO:0005267	122	9	1.57	+	5.73	4.64E-05	2.32E-02	5DPI_iCoV2 + RIBI
**structural molecule activity**	GO:0005198	652	22	8.39	+	2.62	5.56E-05	2.52E-02	5DPI_iCoV2 + RIBI
**potassium ion transmembrane transporter activity**	GO:0015079	160	10	2.06	+	4.86	6.64E-05	2.76E-02	5DPI_iCoV2 + RIBI
**muscle alpha-actinin binding**	GO:0051371	14	4	0.18	+	22.21	6.78E-05	2.6E-02	5DPI_iCoV2 + RIBI

**metal ion transmembrane transporter activity**	GO:0046873	436	17	5.61	+	3.03	7.54E-05	2.51E-02	5DPI_iCoV2 + RIBI
**organic acid binding**	GO:0043177	233	12	3	+	4	7.54E-05	2.69E-02	5DPI_iCoV2 + RIBI
**inorganic cation transmembrane transporter activity**	GO:0022890	533	19	6.86	+	2.77	9.05E-05	2.82E-02	5DPI_iCoV2 + RIBI
**calcium ion binding**	GO:0005509	631	21	8.12	+	2.59	9.89E-05	2.9E-02	5DPI_iCoV2 + RIBI
**FATZ binding**	GO:0051373	5	3	0.06	+	46.64	1.08E-04	3E-02	5DPI_iCoV2 + RIBI
**signaling receptor regulator activity**	GO:0030545	591	20	7.6	+	2.63	1.17E-04	3.07E-02	5DPI_iCoV2 + RIBI
**aryl sulfotransferase activity**	GO:0004062	6	3	0.08	+	38.86	1.61E-04	4.02E-02	5DPI_iCoV2 + RIBI
**sodium channel regulator activity**	GO:0017080	36	5	0.46	+	10.8	1.64E-04	3.91E-02	5DPI_iCoV2 + RIBI
**fatty acid binding**	GO:0005504	60	6	0.77	+	7.77	1.91E-04	4.33E-02	5DPI_iCoV2 + RIBI
**monoatomic cation transmembrane transporter activity**	GO:0008324	572	19	7.36	+	2.58	2.17E-04	4.71E-02	5DPI_iCoV2 + RIBI
**ion binding**	GO:0043167	5378	97	69.19	+	1.4	2.2E-04	4.58E-02	5DPI_iCoV2 + RIBI
**telethonin binding**	GO:0031433	7	3	0.09	+	33.31	2.28E-04	4.55E-02	5DPI_iCoV2 + RIBI
**monoatomic ion gated channel activity**	GO:0022839	315	13	4.05	+	3.21	3.13E-04	6.01E-02	5DPI_iCoV2 + RIBI
**voltage-gated monoatomic ion channel activity**	GO:0005244	197	10	2.53	+	3.95	3.32E-04	6.14E-02	5DPI_iCoV2 + RIBI
**voltage-gated channel activity**	GO:0022832	198	10	2.55	+	3.93	3.45E-04	6.15E-02	5DPI_iCoV2 + RIBI
**gated channel activity**	GO:0022836	320	13	4.12	+	3.16	3.61E-04	6.22E-02	5DPI_iCoV2 + RIBI
**voltage-gated potassium channel activity**	GO:0005249	97	7	1.25	+	5.61	3.68E-04	6.13E-02	5DPI_iCoV2 + RIBI
**inorganic molecular entity transmembrane transporter activity**	GO:0015318	651	20	8.38	+	2.39	5.61E-04	9.03E-02	5DPI_iCoV2 + RIBI
**receptor ligand activity**	GO:0048018	534	17	6.87	+	2.47	7.34E-04	1.14E-01	5DPI_iCoV2 + RIBI
**protein binding**	GO:0005515	9462	150	121.73	+	1.23	8.69E-04	1.31E-01	5DPI_iCoV2 + RIBI
**signaling receptor activator activity**	GO:0030546	545	17	7.01	+	2.42	9.12E-04	1.34E-01	5DPI_iCoV2 + RIBI
**L-threonine ammonia-lyase activity**	GO:0004794	2	2	0.03	+	77.73	9.49E-04	1.25E-01	5DPI_iCoV2 + RIBI
**CCR7 chemokine receptor binding**	GO:0031732	2	2	0.03	+	77.73	9.49E-04	1.28E-01	5DPI_iCoV2 + RIBI
**iron ion transmembrane transporter inhibitor activity**	GO:0097690	2	2	0.03	+	77.73	9.49E-04	1.32E-01	5DPI_iCoV2 + RIBI
**titin Z domain binding**	GO:0070080	2	2	0.03	+	77.73	9.49E-04	1.35E-01	5DPI_iCoV2 + RIBI
**monooxygenase activity**	GO:0004497	151	8	1.94	+	4.12	9.97E-04	1.28E-01	5DPI_iCoV2 + RIBI
**monoatomic ion transmembrane transporter activity**	GO:0015075	700	20	9.01	+	2.22	1.04E-03	1.29E-01	5DPI_iCoV2 + RIBI
**monoatomic cation channel activity**	GO:0005261	322	12	4.14	+	2.9	1.24E-03	1.51E-01	5DPI_iCoV2 + RIBI

**monocarboxylic acid binding**	GO:0033293	88	6	1.13	+	5.3	1.27E-03	1.51E-01	5DPI_iCoV2 + RIBI
**troponin C binding**	GO:0030172	3	2	0.04	+	51.82	1.57E-03	1.78E-01	5DPI_iCoV2 + RIBI
**L-serine ammonia-lyase activity**	GO:0003941	3	2	0.04	+	51.82	1.57E-03	1.82E-01	5DPI_iCoV2 + RIBI
**monoatomic ion channel activity**	GO:0005216	435	14	5.6	+	2.5	1.92E-03	2.13E-01	5DPI_iCoV2 + RIBI
**lipid binding**	GO:0008289	886	23	11.4	+	2.02	2.01E-03	2.18E-01	5DPI_iCoV2 + RIBI
**metal ion binding**	GO:0046872	3634	67	46.75	+	1.43	2.16E-03	2.29E-01	5DPI_iCoV2 + RIBI
**transporter activity**	GO:0005215	1185	28	15.25	+	1.84	2.18E-03	2.27E-01	5DPI_iCoV2 + RIBI
**salt transmembrane transporter activity**	GO:1901702	582	17	7.49	+	2.27	2.23E-03	2.27E-01	5DPI_iCoV2 + RIBI
**transmembrane receptor protein serine/threonine kinase activity**	GO:0004675	18	3	0.23	+	12.95	2.28E-03	2.27E-01	5DPI_iCoV2 + RIBI
**oleic acid binding**	GO:0070538	4	2	0.05	+	38.86	2.33E-03	2.28E-01	5DPI_iCoV2 + RIBI
**ion channel regulator activity**	GO:0099106	136	7	1.75	+	4	2.39E-03	2.29E-01	5DPI_iCoV2 + RIBI
**odorant binding**	GO:0005549	534	0	6.87	-	< 0.01	2.41E-03	2.27E-01	5DPI_iCoV2 + RIBI
**hormone activity**	GO:0005179	137	7	1.76	+	3.97	2.48E-03	2.29E-01	5DPI_iCoV2 + RIBI
**long-chain fatty acid transporter activity**	GO:0005324	19	3	0.24	+	12.27	2.61E-03	2.37E-01	5DPI_iCoV2 + RIBI
**actin filament binding**	GO:0051015	219	9	2.82	+	3.19	2.65E-03	2.36E-01	5DPI_iCoV2 + RIBI
**scavenger receptor activity**	GO:0005044	43	4	0.55	+	7.23	2.96E-03	2.59E-01	5DPI_iCoV2 + RIBI
**BMP binding**	GO:0036122	20	3	0.26	+	11.66	2.97E-03	2.56E-01	5DPI_iCoV2 + RIBI
**voltage-gated monoatomic cation channel activity**	GO:0022843	143	7	1.84	+	3.8	3.12E-03	2.64E-01	5DPI_iCoV2 + RIBI
**structural constituent of cytoskeleton**	GO:0005200	73	5	0.94	+	5.32	3.17E-03	2.63E-01	5DPI_iCoV2 + RIBI
**ammonia-lyase activity**	GO:0016841	5	2	0.06	+	31.09	3.24E-03	2.65E-01	5DPI_iCoV2 + RIBI
**long-chain fatty acid binding**	GO:0036041	21	3	0.27	+	11.1	3.37E-03	2.71E-01	5DPI_iCoV2 + RIBI
**channel activity**	GO:0015267	495	15	6.37	+	2.36	3.72E-03	2.9E-01	5DPI_iCoV2 + RIBI
**passive transmembrane transporter activity**	GO:0022803	495	15	6.37	+	2.36	3.72E-03	2.95E-01	5DPI_iCoV2 + RIBI
**C-acyltransferase activity**	GO:0016408	22	3	0.28	+	10.6	3.79E-03	2.91E-01	5DPI_iCoV2 + RIBI
**cation binding**	GO:0043169	3732	67	48.01	+	1.4	4.08E-03	3.09E-01	5DPI_iCoV2 + RIBI
**fatty-acyl-CoA synthase activity**	GO:0004321	6	2	0.08	+	25.91	4.28E-03	3.1E-01	5DPI_iCoV2 + RIBI
**metal ion sequestering activity**	GO:0140487	6	2	0.08	+	25.91	4.28E-03	3.14E-01	5DPI_iCoV2 + RIBI
**dinitrosyl-iron complex binding**	GO:0035731	6	2	0.08	+	25.91	4.28E-03	3.19E-01	5DPI_iCoV2 + RIBI
**signaling receptor binding**	GO:0005102	1650	35	21.23	+	1.65	4.31E-03	3.07E-01	5DPI_iCoV2 + RIBI

**channel regulator activity**	GO:0016247	154	7	1.98	+	3.53	4.6E-03	3.23E-01	5DPI_iCoV2 + RIBI
**transferase activity, transferring sulphur-containing groups**	GO:0016782	81	5	1.04	+	4.8	4.81E-03	3.34E-01	5DPI_iCoV2 + RIBI
**cargo receptor activity**	GO:0038024	82	5	1.05	+	4.74	5.05E-03	3.46E-01	5DPI_iCoV2 + RIBI
**transferase activity**	GO:0016740	2239	44	28.81	+	1.53	5.44E-03	3.67E-01	5DPI_iCoV2 + RIBI
**extracellularly glutamate-gated ion channel activity**	GO:0005234	7	2	0.09	+	22.21	5.46E-03	3.54E-01	5DPI_iCoV2 + RIBI
**BMP receptor activity**	GO:0098821	7	2	0.09	+	22.21	5.46E-03	3.58E-01	5DPI_iCoV2 + RIBI
**medium-chain fatty acid-CoA ligase activity**	GO:0031956	7	2	0.09	+	22.21	5.46E-03	3.63E-01	5DPI_iCoV2 + RIBI
**sodium ion transmembrane transporter activity**	GO:0015081	160	7	2.06	+	3.4	5.6E-03	3.58E-01	5DPI_iCoV2 + RIBI
**delayed rectifier potassium channel activity**	GO:0005251	26	3	0.33	+	8.97	5.8E-03	3.62E-01	5DPI_iCoV2 + RIBI
**actin monomer binding**	GO:0003785	26	3	0.33	+	8.97	5.8E-03	3.67E-01	5DPI_iCoV2 + RIBI
**transmembrane transporter binding**	GO:0044325	162	7	2.08	+	3.36	5.97E-03	3.68E-01	5DPI_iCoV2 + RIBI
**sulfur compound binding**	GO:1901681	297	10	3.82	+	2.62	6.12E-03	3.73E-01	5DPI_iCoV2 + RIBI
**transmembrane signaling receptor activity**	GO:0004888	2203	15	28.34	-	0.53	6.69E-03	4.02E-01	5DPI_iCoV2 + RIBI
**NAD+-protein-arginine ADP-ribosyltransferase activity**	GO:0106274	8	2	0.1	+	19.43	6.76E-03	3.97E-01	5DPI_iCoV2 + RIBI
**D-glucose transmembrane transporter activity**	GO:0055056	8	2	0.1	+	19.43	6.76E-03	4.02E-01	5DPI_iCoV2 + RIBI
**lipase activity**	GO:0016298	127	6	1.63	+	3.67	7.13E-03	4.14E-01	5DPI_iCoV2 + RIBI
**identical protein binding**	GO:0042802	2410	46	31.01	+	1.48	7.2E-03	4.13E-01	5DPI_iCoV2 + RIBI
**uniporter activity**	GO:0015292	9	2	0.12	+	17.27	8.2E-03	4.6E-01	5DPI_iCoV2 + RIBI
**butyrate-CoA ligase activity**	GO:0047760	9	2	0.12	+	17.27	8.2E-03	4.65E-01	5DPI_iCoV2 + RIBI
**sulfotransferase activity**	GO:0008146	61	4	0.78	+	5.1	9.41E-03	5.22E-01	5DPI_iCoV2 + RIBI
**heparin binding**	GO:0008201	178	7	2.29	+	3.06	9.59E-03	5.26E-01	5DPI_iCoV2 + RIBI
**alcohol sulfotransferase activity**	GO:0004027	10	2	0.13	+	15.55	9.76E-03	5.29E-01	5DPI_iCoV2 + RIBI
**steroid delta-isomerase activity**	GO:0004769	11	2	0.14	+	14.13	1.14E-02	6.14E-01	5DPI_iCoV2 + RIBI
**cytokine binding**	GO:0019955	142	6	1.83	+	3.28	1.17E-02	6.22E-01	5DPI_iCoV2 + RIBI
**extracellular matrix structural constituent**	GO:0005201	145	6	1.87	+	3.22	1.28E-02	6.75E-01	5DPI_iCoV2 + RIBI
**myosin heavy chain binding**	GO:0032036	12	2	0.15	+	12.95	1.32E-02	6.81E-01	5DPI_iCoV2 + RIBI
**aromatase activity**	GO:0070330	36	3	0.46	+	6.48	1.32E-02	6.88E-01	5DPI_iCoV2 + RIBI
**microfilament motor activity**	GO:0000146	37	3	0.48	+	6.3	1.42E-02	7.22E-01	5DPI_iCoV2 + RIBI
**glycosaminoglycan binding**	GO:0005539	240	8	3.09	+	2.59	1.43E-02	7.14E-01	5DPI_iCoV2 + RIBI

**lyase activity**	GO:0016829	193	7	2.48	+	2.82	1.43E-02	7.19E-01	5DPI_iCoV2 + RIBI
**oxidoreductase activity**	GO:0016491	769	18	9.89	+	1.82	1.45E-02	7.11E-01	5DPI_iCoV2 + RIBI
**growth factor activity**	GO:0008083	149	6	1.92	+	3.13	1.45E-02	7.14E-01	5DPI_iCoV2 + RIBI
**metal cation:monoatomic cation antiporter activity**	GO:0140828	38	3	0.49	+	6.14	1.51E-02	7.2E-01	5DPI_iCoV2 + RIBI
**azole transmembrane transporter activity**	GO:1901474	13	2	0.17	+	11.96	1.51E-02	7.26E-01	5DPI_iCoV2 + RIBI
**carbon-nitrogen lyase activity**	GO:0016840	13	2	0.17	+	11.96	1.51E-02	7.33E-01	5DPI_iCoV2 + RIBI
**nucleic acid binding**	GO:0003676	3371	29	43.37	-	0.67	1.58E-02	7.45E-01	5DPI_iCoV2 + RIBI
**L-glutamate transmembrane transporter activity**	GO:0005313	14	2	0.18	+	11.1	1.72E-02	7.93E-01	5DPI_iCoV2 + RIBI
**tropomyosin binding**	GO:0005523	14	2	0.18	+	11.1	1.72E-02	8E-01	5DPI_iCoV2 + RIBI
**transmembrane transporter activity**	GO:0022857	1077	23	13.86	+	1.66	1.81E-02	8.31E-01	5DPI_iCoV2 + RIBI
**transmembrane receptor protein kinase activity**	GO:0019199	76	4	0.98	+	4.09	1.9E-02	8.61E-01	5DPI_iCoV2 + RIBI
**RNA binding**	GO:0003723	1113	6	14.32	-	0.42	1.94E-02	8.71E-01	5DPI_iCoV2 + RIBI
**catalytic activity**	GO:0003824	5563	89	71.57	+	1.24	1.95E-02	8.67E-01	5DPI_iCoV2 + RIBI
**oxidoreductase activity, acting on paired donors, with incorporation or reduction of molecular oxygen, reduced flavin or flavoprotein as one donor, and incorporation of one atom of oxygen**	GO:0016712	78	4	1	+	3.99	2.06E-02	9.09E-01	5DPI_iCoV2 + RIBI
**signaling receptor activity**	GO:0038023	2418	19	31.11	-	0.61	2.1E-02	9.1E-01	5DPI_iCoV2 + RIBI
**molecular transducer activity**	GO:0060089	2418	19	31.11	-	0.61	2.1E-02	9.18E-01	5DPI_iCoV2 + RIBI
**sodium channel activity**	GO:0005272	44	3	0.57	+	5.3	2.18E-02	9.38E-01	5DPI_iCoV2 + RIBI
**carboxylic ester hydrolase activity**	GO:0052689	165	6	2.12	+	2.83	2.23E-02	9.51E-01	5DPI_iCoV2 + RIBI
**voltage-gated monoatomic ion channel activity involved in regulation of presynaptic membrane potential**	GO:0099508	17	2	0.22	+	9.14	2.38E-02	1E+00	5DPI_iCoV2 + RIBI
**amide transmembrane transporter activity**	GO:0042887	46	3	0.59	+	5.07	2.43E-02	1E+00	5DPI_iCoV2 + RIBI
**ubiquitin protein ligase binding**	GO:0031625	335	0	4.31	-	< 0.01	2.43E-02	1E+00	5DPI_iCoV2 + RIBI
**protein homodimerization activity**	GO:0042803	803	18	10.33	+	1.74	2.45E-02	1E+00	5DPI_iCoV2 + RIBI
**L-asparagine, sodium:proton antiporter activity**	GO:0140831	1	1	0.01	+	77.73	2.52E-02	8.4E-01	5DPI_iCoV2 + RIBI
**L-histidine, sodium:proton antiporter activity**	GO:0140832	1	1	0.01	+	77.73	2.52E-02	8.46E-01	5DPI_iCoV2 + RIBI
**L-tyrosine:2-oxoglutarate aminotransferase activity**	GO:0004838	1	1	0.01	+	77.73	2.52E-02	8.51E-01	5DPI_iCoV2 + RIBI
**asialoglycoprotein receptor activity**	GO:0004873	1	1	0.01	+	77.73	2.52E-02	8.57E-01	5DPI_iCoV2 + RIBI
**insulin-responsive glucose:proton symporter activity**	GO:0005360	1	1	0.01	+	77.73	2.52E-02	8.63E-01	5DPI_iCoV2 + RIBI

**10-hydroxy-9-octadecanoate phosphatase activity**	GO:0033885	1	1	0.01	+	77.73	2.52E-02	8.69E-01	5DPI_iCoV2 + RIBI
**mast cell secretagogue receptor activity**	GO:1990595	1	1	0.01	+	77.73	2.52E-02	8.75E-01	5DPI_iCoV2 + RIBI
**thyroid-stimulating hormone receptor activity**	GO:0004996	1	1	0.01	+	77.73	2.52E-02	8.81E-01	5DPI_iCoV2 + RIBI
**interleukin-22 binding**	GO:0042017	1	1	0.01	+	77.73	2.52E-02	8.87E-01	5DPI_iCoV2 + RIBI
**glucose uniporter activity**	GO:0015304	1	1	0.01	+	77.73	2.52E-02	8.94E-01	5DPI_iCoV2 + RIBI
**sodium:inorganic phosphate symporter activity**	GO:0015319	1	1	0.01	+	77.73	2.52E-02	9E-01	5DPI_iCoV2 + RIBI
**L-tyrosine aminotransferase activity**	GO:0070547	1	1	0.01	+	77.73	2.52E-02	9.06E-01	5DPI_iCoV2 + RIBI
**hydroperoxy icosatetraenoate isomerase activity**	GO:0106255	1	1	0.01	+	77.73	2.52E-02	9.13E-01	5DPI_iCoV2 + RIBI
**protein serine kinase activity**	GO:0106264	1	1	0.01	+	77.73	2.52E-02	9.2E-01	5DPI_iCoV2 + RIBI
**secondary active organic cation transmembrane transporter activity**	GO:0008513	1	1	0.01	+	77.73	2.52E-02	9.26E-01	5DPI_iCoV2 + RIBI
**omega-hydroxyceramide transacylase activity**	GO:0106341	1	1	0.01	+	77.73	2.52E-02	9.33E-01	5DPI_iCoV2 + RIBI
**azole:proton antiporter activity**	GO:0045119	1	1	0.01	+	77.73	2.52E-02	9.4E-01	5DPI_iCoV2 + RIBI
**voltage-gated potassium channel activity involved in bundle of His cell action potential repolarization**	GO:0086087	1	1	0.01	+	77.73	2.52E-02	9.47E-01	5DPI_iCoV2 + RIBI
**voltage-gated potassium channel activity involved in SA node cell action potential repolarization**	GO:0086090	1	1	0.01	+	77.73	2.52E-02	9.54E-01	5DPI_iCoV2 + RIBI
**glycerophosphoinositol inositolphosphodiesterase activity**	GO:0047394	1	1	0.01	+	77.73	2.52E-02	9.62E-01	5DPI_iCoV2 + RIBI
**L-gulonolactone oxidase activity**	GO:0050105	1	1	0.01	+	77.73	2.52E-02	9.69E-01	5DPI_iCoV2 + RIBI
**D-arabinono-1,4-lactone oxidase activity**	GO:0003885	1	1	0.01	+	77.73	2.52E-02	9.77E-01	5DPI_iCoV2 + RIBI
**calcium ion sequestering activity**	GO:0140314	1	1	0.01	+	77.73	2.52E-02	9.84E-01	5DPI_iCoV2 + RIBI
**4-nitrophenol 2-monooxygenase activity**	GO:0018601	1	1	0.01	+	77.73	2.52E-02	9.92E-01	5DPI_iCoV2 + RIBI
**glycosylphosphatidylinositol phospholipase D activity**	GO:0004621	1	1	0.01	+	77.73	2.52E-02	1E+00	5DPI_iCoV2 + RIBI
**intramolecular transferase activity, transferring hydroxy groups**	GO:0050486	1	1	0.01	+	77.73	2.52E-02	1E+00	5DPI_iCoV2 + RIBI
**propionate CoA-transferase activity**	GO:0018729	1	1	0.01	+	77.73	2.52E-02	1E+00	5DPI_iCoV2 + RIBI
**thiol S-methyltransferase activity**	GO:0018708	1	1	0.01	+	77.73	2.52E-02	1E+00	5DPI_iCoV2 + RIBI
**albendazole monooxygenase activity**	GO:0047638	1	1	0.01	+	77.73	2.52E-02	1E+00	5DPI_iCoV2 + RIBI
**ubiquitin-like protein ligase binding**	GO:0044389	352	0	4.53	-	< 0.01	2.57E-02	8.5E-01	5DPI_iCoV2 + RIBI
**neurotransmitter transmembrane transporter activity**	GO:0005326	18	2	0.23	+	8.64	2.63E-02	8.52E-01	5DPI_iCoV2 + RIBI

**transmembrane receptor protein tyrosine kinase activator activity**	GO:0030297	18	2	0.23	+	8.64	2.63E-02	8.57E-01	5DPI_iCoV2 + RIBI
**glutathione binding**	GO:0043295	18	2	0.23	+	8.64	2.63E-02	8.63E-01	5DPI_iCoV2 + RIBI
**molecular_function**	GO:0003674	20579	274	264.76	+	1.03	2.67E-02	8.53E-01	5DPI_iCoV2 + RIBI
**Unclassified**	UNCLASSIFIED	1418	9	18.24	-	0.49	2.67E-02	8.59E-01	5DPI_iCoV2 + RIBI
**arachidonic acid epoxygenase activity**	GO:0008392	48	3	0.62	+	4.86	2.7E-02	8.58E-01	5DPI_iCoV2 + RIBI
**antioxidant activity**	GO:0016209	86	4	1.11	+	3.62	2.78E-02	8.79E-01	5DPI_iCoV2 + RIBI
**triglyceride lipase activity**	GO:0004806	19	2	0.24	+	8.18	2.88E-02	8.98E-01	5DPI_iCoV2 + RIBI
**acidic amino acid transmembrane transporter activity**	GO:0015172	19	2	0.24	+	8.18	2.88E-02	9.04E-01	5DPI_iCoV2 + RIBI
**scaffold protein binding**	GO:0097110	89	4	1.15	+	3.49	3.09E-02	9.57E-01	5DPI_iCoV2 + RIBI
**arachidonic acid monooxygenase activity**	GO:0008391	51	3	0.66	+	4.57	3.13E-02	9.63E-01	5DPI_iCoV2 + RIBI
**oxidoreductase activity, acting on paired donors, with incorporation or reduction of molecular oxygen**	GO:0016705	229	7	2.95	+	2.38	3.16E-02	9.69E-01	5DPI_iCoV2 + RIBI
**lipid transporter activity**	GO:0005319	181	6	2.33	+	2.58	3.26E-02	9.92E-01	5DPI_iCoV2 + RIBI
**acyltransferase activity, transferring groups other than amino-acyl groups**	GO:0016747	231	7	2.97	+	2.36	3.29E-02	9.95E-01	5DPI_iCoV2 + RIBI
**calcium-dependent protein binding**	GO:0048306	91	4	1.17	+	3.42	3.3E-02	9.93E-01	5DPI_iCoV2 + RIBI
**anion binding**	GO:0043168	2371	42	30.5	+	1.38	3.38E-02	1E+00	5DPI_iCoV2 + RIBI
**fatty acid ligase activity**	GO:0015645	21	2	0.27	+	7.4	3.41E-02	1E+00	5DPI_iCoV2 + RIBI
**pyridoxal phosphate binding**	GO:0030170	53	3	0.68	+	4.4	3.43E-02	1E+00	5DPI_iCoV2 + RIBI
**vitamin B6 binding**	GO:0070279	54	3	0.69	+	4.32	3.59E-02	1E+00	5DPI_iCoV2 + RIBI
**protein heterodimerization activity**	GO:0046982	303	0	3.9	-	< 0.01	3.61E-02	1E+00	5DPI_iCoV2 + RIBI
**phosphatidylinositol-5-phosphate binding**	GO:0010314	22	2	0.28	+	7.07	3.69E-02	1E+00	5DPI_iCoV2 + RIBI
**intramolecular oxidoreductase activity, transposing C=C bonds**	GO:0016863	22	2	0.28	+	7.07	3.69E-02	1E+00	5DPI_iCoV2 + RIBI
**oxidoreductase activity, acting on paired donors, with incorporation or reduction of molecular oxygen, NADH as one donor, and incorporation of one atom of oxygen**	GO:0016709	55	3	0.71	+	4.24	3.75E-02	1E+00	5DPI_iCoV2 + RIBI
**phosphate ion uniporter activity**	GO:0140787	2	1	0.03	+	38.86	3.76E-02	9.48E-01	5DPI_iCoV2 + RIBI
**ferritin receptor activity**	GO:0070287	2	1	0.03	+	38.86	3.76E-02	9.53E-01	5DPI_iCoV2 + RIBI
**L-glutamine, sodium:proton antiporter activity**	GO:0140830	2	1	0.03	+	38.86	3.76E-02	9.58E-01	5DPI_iCoV2 + RIBI
**hepoxilin A3 synthase activity**	GO:0051120	2	1	0.03	+	38.86	3.76E-02	9.63E-01	5DPI_iCoV2 + RIBI

**interleukin-8 receptor activity**	GO:0004918	2	1	0.03	+	38.86	3.76E-02	9.68E-01	5DPI_iCoV2 + RIBI
**extracellularly glutamate-gated chloride channel activity**	GO:0008068	2	1	0.03	+	38.86	3.76E-02	9.73E-01	5DPI_iCoV2 + RIBI
**corticotropin hormone receptor binding**	GO:0031780	2	1	0.03	+	38.86	3.76E-02	9.78E-01	5DPI_iCoV2 + RIBI
**type 5 melanocortin receptor binding**	GO:0031783	2	1	0.03	+	38.86	3.76E-02	9.83E-01	5DPI_iCoV2 + RIBI
**chemoattractant activity involved in axon guidance**	GO:1902379	2	1	0.03	+	38.86	3.76E-02	9.88E-01	5DPI_iCoV2 + RIBI
**coumarin 7-hydroxylase activity**	GO:0008389	2	1	0.03	+	38.86	3.76E-02	9.94E-01	5DPI_iCoV2 + RIBI
**monoatomic ion antiporter activity involved in regulation of postsynaptic membrane potential**	GO:0099580	2	1	0.03	+	38.86	3.76E-02	9.99E-01	5DPI_iCoV2 + RIBI
**phosphoenolpyruvate carboxykinase activity**	GO:0004613	2	1	0.03	+	38.86	3.76E-02	1E+00	5DPI_iCoV2 + RIBI
**phosphoenolpyruvate carboxykinase activity**	GO:0004611	2	1	0.03	+	38.86	3.76E-02	1E+00	5DPI_iCoV2 + RIBI
**tryptophan 5-monooxygenase activity**	GO:0004510	2	1	0.03	+	38.86	3.76E-02	1E+00	5DPI_iCoV2 + RIBI
**zinc ion sequestering activity**	GO:0140486	2	1	0.03	+	38.86	3.76E-02	1E+00	5DPI_iCoV2 + RIBI
**activin receptor antagonist activity**	GO:0038102	2	1	0.03	+	38.86	3.76E-02	1E+00	5DPI_iCoV2 + RIBI
**1-alpha,25-dihydroxyvitamin D3 23-hydroxylase activity**	GO:0062181	2	1	0.03	+	38.86	3.76E-02	1E+00	5DPI_iCoV2 + RIBI
**vitamin D 23-hydroxylase activity**	GO:0062179	2	1	0.03	+	38.86	3.76E-02	1E+00	5DPI_iCoV2 + RIBI
**ADP-ribosylarginine hydrolase activity**	GO:0003875	2	1	0.03	+	38.86	3.76E-02	1E+00	5DPI_iCoV2 + RIBI
**acyl-coenzyme A diphosphatase activity**	GO:0106399	2	1	0.03	+	38.86	3.76E-02	1E+00	5DPI_iCoV2 + RIBI
**trimethylamine monooxygenase activity**	GO:0034899	2	1	0.03	+	38.86	3.76E-02	1E+00	5DPI_iCoV2 + RIBI
**glucosaminylgalactosylglucosylceramide beta-galactosyltransferase activity**	GO:0047275	2	1	0.03	+	38.86	3.76E-02	1E+00	5DPI_iCoV2 + RIBI
**hexose uniporter activity**	GO:0008516	2	1	0.03	+	38.86	3.76E-02	1E+00	5DPI_iCoV2 + RIBI
**aryldialkylphosphatase activity**	GO:0004063	2	1	0.03	+	38.86	3.76E-02	1E+00	5DPI_iCoV2 + RIBI
**catalytic activity, acting on a nucleic acid**	GO:0140640	588	2	7.56	-	0.26	3.79E-02	9.46E-01	5DPI_iCoV2 + RIBI
**ribonucleoside triphosphate phosphatase activity**	GO:0017111	587	2	7.55	-	0.26	3.79E-02	9.49E-01	5DPI_iCoV2 + RIBI
**phosphatidic acid binding**	GO:0070300	23	2	0.3	+	6.76	3.98E-02	9.79E-01	5DPI_iCoV2 + RIBI
**ankyrin binding**	GO:0030506	23	2	0.3	+	6.76	3.98E-02	9.84E-01	5DPI_iCoV2 + RIBI
**NAD+ ADP-ribosyltransferase activity**	GO:0003950	23	2	0.3	+	6.76	3.98E-02	9.88E-01	5DPI_iCoV2 + RIBI
**hydro-lyase activity**	GO:0016836	58	3	0.75	+	4.02	4.26E-02	1E+00	5DPI_iCoV2 + RIBI
**metal cation:proton antiporter activity**	GO:0051139	24	2	0.31	+	6.48	4.28E-02	1E+00	5DPI_iCoV2 + RIBI
**NAD+-protein ADP-ribosyltransferase activity**	GO:1990404	24	2	0.31	+	6.48	4.28E-02	1E+00	5DPI_iCoV2 + RIBI

**serine-type endopeptidase activity**	GO:0004252	194	6	2.5	+	2.4	4.29E-02	1E+00	5DPI_iCoV2 + RIBI
**calmodulin binding**	GO:0005516	194	6	2.5	+	2.4	4.29E-02	1E+00	5DPI_iCoV2 + RIBI
**hexose transmembrane transporter activity**	GO:0015149	25	2	0.32	+	6.22	4.58E-02	1E+00	5DPI_iCoV2 + RIBI
**glucose transmembrane transporter activity**	GO:0005355	25	2	0.32	+	6.22	4.58E-02	1E+00	5DPI_iCoV2 + RIBI
**intramolecular transferase activity**	GO:0016866	25	2	0.32	+	6.22	4.58E-02	1E+00	5DPI_iCoV2 + RIBI
**voltage-gated sodium channel activity**	GO:0005248	25	2	0.32	+	6.22	4.58E-02	1E+00	5DPI_iCoV2 + RIBI
**protein kinase C binding**	GO:0005080	60	3	0.77	+	3.89	4.61E-02	1E+00	5DPI_iCoV2 + RIBI
**symporter activity**	GO:0015293	150	5	1.93	+	2.59	4.81E-02	1E+00	5DPI_iCoV2 + RIBI
**glutathione peroxidase activity**	GO:0004602	26	2	0.33	+	5.98	4.89E-02	1E+00	5DPI_iCoV2 + RIBI
**CoA-ligase activity**	GO:0016405	26	2	0.33	+	5.98	4.89E-02	1E+00	5DPI_iCoV2 + RIBI
**phosphoglycerate mutase activity**	GO:0004619	3	1	0.04	+	25.91	4.99E-02	1E+00	5DPI_iCoV2 + RIBI
**nitrate reductase activity**	GO:0008940	3	1	0.04	+	25.91	4.99E-02	1E+00	5DPI_iCoV2 + RIBI
**alpha-amylase activity**	GO:0004556	3	1	0.04	+	25.91	4.99E-02	1E+00	5DPI_iCoV2 + RIBI
**adenosine-phosphate deaminase activity**	GO:0047623	3	1	0.04	+	25.91	4.99E-02	1E+00	5DPI_iCoV2 + RIBI
**nitric-oxide synthase activity**	GO:0004517	3	1	0.04	+	25.91	4.99E-02	1E+00	5DPI_iCoV2 + RIBI
**retinol O-fatty-acyltransferase activity**	GO:0050252	3	1	0.04	+	25.91	4.99E-02	1E+00	5DPI_iCoV2 + RIBI
**neuregulin receptor activity**	GO:0038131	3	1	0.04	+	25.91	4.99E-02	1E+00	5DPI_iCoV2 + RIBI
**G protein-coupled pyrimidinergic nucleotide receptor activity**	GO:0071553	3	1	0.04	+	25.91	4.99E-02	1E+00	5DPI_iCoV2 + RIBI
**glycogen synthase activity**	GO:0004373	3	1	0.04	+	25.91	4.99E-02	1E+00	5DPI_iCoV2 + RIBI
**AMP deaminase activity**	GO:0003876	3	1	0.04	+	25.91	4.99E-02	1E+00	5DPI_iCoV2 + RIBI
**acetylcholine receptor activator activity**	GO:0030549	3	1	0.04	+	25.91	4.99E-02	1E+00	5DPI_iCoV2 + RIBI
**acyl-L-homoserine-lactone lactonohydrolase activity**	GO:0102007	3	1	0.04	+	25.91	4.99E-02	1E+00	5DPI_iCoV2 + RIBI
**voltage-gated potassium channel activity involved in atrial cardiac muscle cell action potential repolarization**	GO:0086089	3	1	0.04	+	25.91	4.99E-02	1E+00	5DPI_iCoV2 + RIBI
**zymogen binding**	GO:0035375	3	1	0.04	+	25.91	4.99E-02	1E+00	5DPI_iCoV2 + RIBI
**G protein-coupled UTP receptor activity**	GO:0045030	3	1	0.04	+	25.91	4.99E-02	1E+00	5DPI_iCoV2 + RIBI
**spermidine transmembrane transporter activity**	GO:0015606	3	1	0.04	+	25.91	4.99E-02	1E+00	5DPI_iCoV2 + RIBI
**vitamin D3 25-hydroxylase activity**	GO:0030343	3	1	0.04	+	25.91	4.99E-02	1E+00	5DPI_iCoV2 + RIBI
**bisphosphoglycerate mutase activity**	GO:0004082	3	1	0.04	+	25.91	4.99E-02	1E+00	5DPI_iCoV2 + RIBI

**interleukin-8 binding**	GO:0019959	3	1	0.04	+	25.91	4.99E-02	1E+00	5DPI_iCoV2 + RIBI
**calcium:monoatomic cation antiporter activity involved in regulation of postsynaptic cytosolic calcium ion concentration**	GO:1905060	3	1	0.04	+	25.91	4.99E-02	1E+00	5DPI_iCoV2 + RIBI
**lactonohydrolase activity**	GO:0046573	3	1	0.04	+	25.91	4.99E-02	1E+00	5DPI_iCoV2 + RIBI
**2,3-bisphosphoglycerate-dependent phosphoglycerate mutase activity**	GO:0046538	3	1	0.04	+	25.91	4.99E-02	1E+00	5DPI_iCoV2 + RIBI
**vitamin D 24-hydroxylase activity**	GO:0070576	3	1	0.04	+	25.91	4.99E-02	1E+00	5DPI_iCoV2 + RIBI
**interleukin-22 receptor activity**	GO:0042018	3	1	0.04	+	25.91	4.99E-02	1E+00	5DPI_iCoV2 + RIBI
**CCR10 chemokine receptor binding**	GO:0031735	3	1	0.04	+	25.91	4.99E-02	1E+00	5DPI_iCoV2 + RIBI
**glucose:proton symporter activity**	GO:0005356	3	1	0.04	+	25.91	4.99E-02	1E+00	5DPI_iCoV2 + RIBI
**norepinephrine:sodium symporter activity**	GO:0005334	3	1	0.04	+	25.91	4.99E-02	1E+00	5DPI_iCoV2 + RIBI
**hexose:proton symporter activity**	GO:0009679	3	1	0.04	+	25.91	4.99E-02	1E+00	5DPI_iCoV2 + RIBI
**acetylcholine transmembrane transporter activity**	GO:0005277	3	1	0.04	+	25.91	4.99E-02	1E+00	5DPI_iCoV2 + RIBI
**L-glutamate uniporter activity**	GO:0140788	3	1	0.04	+	25.91	4.99E-02	1E+00	5DPI_iCoV2 + RIBI
**acetate ester transmembrane transporter activity**	GO:1901375	3	1	0.04	+	25.91	4.99E-02	1E+00	5DPI_iCoV2 + RIBI
**cytokine activity**	GO:0005125	231	28	2.36	+	11.85	9.15E-21	4.56E-17	5DPI_iCoV2 + Alum
**receptor ligand activity**	GO:0048018	534	35	5.46	+	6.41	9.58E-18	2.39E-14	5DPI_iCoV2 + Alum
**chemokine activity**	GO:0008009	42	15	0.43	+	34.92	1.14E-17	1.9E-14	5DPI_iCoV2 + Alum
**signaling receptor activator activity**	GO:0030546	545	35	5.57	+	6.28	1.74E-17	2.17E-14	5DPI_iCoV2 + Alum
**signaling receptor regulator activity**	GO:0030545	591	36	6.05	+	5.96	2.8E-17	2.8E-14	5DPI_iCoV2 + Alum
**chemokine receptor binding**	GO:0042379	79	15	0.81	+	18.56	3.46E-14	2.87E-11	5DPI_iCoV2 + Alum
**cytokine receptor binding**	GO:0005126	316	23	3.23	+	7.12	7.36E-13	5.25E-10	5DPI_iCoV2 + Alum
**molecular function regulator activity**	GO:0098772	2147	59	21.96	+	2.69	1.94E-12	1.21E-09	5DPI_iCoV2 + Alum
**molecular function activator activity**	GO:0140677	1192	41	12.19	+	3.36	1.43E-11	7.91E-09	5DPI_iCoV2 + Alum
**CCR chemokine receptor binding**	GO:0048020	58	11	0.59	+	18.54	9.78E-11	4.88E-08	5DPI_iCoV2 + Alum
**signaling receptor binding**	GO:0005102	1650	47	16.88	+	2.78	1.91E-10	8.68E-08	5DPI_iCoV2 + Alum
**G protein-coupled receptor binding**	GO:0001664	328	17	3.36	+	5.07	9.44E-08	3.93E-05	5DPI_iCoV2 + Alum
**endopeptidase activity**	GO:0004175	453	19	4.63	+	4.1	3.79E-07	1.45E-04	5DPI_iCoV2 + Alum
**growth factor activity**	GO:0008083	149	11	1.52	+	7.22	7.43E-07	2.65E-04	5DPI_iCoV2 + Alum
**serine-type endopeptidase activity**	GO:0004252	194	12	1.98	+	6.05	1.36E-06	4.52E-04	5DPI_iCoV2 + Alum

**serine-type peptidase activity**	GO:0008236	211	12	2.16	+	5.56	3.1E-06	9.67E-04	5DPI_iCoV2 + Alum
**serine hydrolase activity**	GO:0017171	216	12	2.21	+	5.43	3.9E-06	1.14E-03	5DPI_iCoV2 + Alum
**peptidase activity**	GO:0008233	642	21	6.57	+	3.2	4.27E-06	1.18E-03	5DPI_iCoV2 + Alum
**peptidase regulator activity**	GO:0061134	267	13	2.73	+	4.76	6.07E-06	1.59E-03	5DPI_iCoV2 + Alum
**endopeptidase regulator activity**	GO:0061135	214	11	2.19	+	5.03	1.97E-05	4.93E-03	5DPI_iCoV2 + Alum
**CXCR chemokine receptor binding**	GO:0045236	15	4	0.15	+	26.07	3.52E-05	8.37E-03	5DPI_iCoV2 + Alum
**endopeptidase inhibitor activity**	GO:0004866	195	10	1.99	+	5.01	4.81E-05	1.09E-02	5DPI_iCoV2 + Alum
**carbohydrate binding**	GO:0030246	283	12	2.89	+	4.15	5.08E-05	1.1E-02	5DPI_iCoV2 + Alum
**peptidase inhibitor activity**	GO:0030414	202	10	2.07	+	4.84	6.38E-05	1.33E-02	5DPI_iCoV2 + Alum
**CCR1 chemokine receptor binding**	GO:0031726	7	3	0.07	+	41.9	1.17E-04	2.33E-02	5DPI_iCoV2 + Alum
**olfactory receptor activity**	GO:0004984	1169	1	11.96	-	0.08	1.24E-04	2.38E-02	5DPI_iCoV2 + Alum
**chitin binding**	GO:0008061	11	3	0.11	+	26.66	3.43E-04	6.35E-02	5DPI_iCoV2 + Alum
**extracellular matrix binding**	GO:0050840	57	5	0.58	+	8.58	4.12E-04	7.34E-02	5DPI_iCoV2 + Alum
**cytokine receptor activity**	GO:0004896	95	6	0.97	+	6.17	5.74E-04	9.88E-02	5DPI_iCoV2 + Alum
**purine ribonucleoside triphosphate binding**	GO:0035639	1733	5	17.73	-	0.28	6.28E-04	1.04E-01	5DPI_iCoV2 + Alum
**immune receptor activity**	GO:0140375	137	7	1.4	+	5	6.76E-04	1.09E-01	5DPI_iCoV2 + Alum
**fibronectin binding**	GO:0001968	36	4	0.37	+	10.86	7.03E-04	1.1E-01	5DPI_iCoV2 + Alum
**purine ribonucleotide binding**	GO:0032555	1814	6	18.55	-	0.32	8.82E-04	1.33E-01	5DPI_iCoV2 + Alum
**collagen binding**	GO:0005518	70	5	0.72	+	6.98	9.88E-04	1.45E-01	5DPI_iCoV2 + Alum
**nucleotide binding**	GO:0000166	2079	8	21.27	-	0.38	1.18E-03	1.64E-01	5DPI_iCoV2 + Alum
**nucleoside phosphate binding**	GO:1901265	2079	8	21.27	-	0.38	1.18E-03	1.68E-01	5DPI_iCoV2 + Alum
**arachidonate 12-lipoxygenase activity**	GO:0004052	4	2	0.04	+	48.88	1.49E-03	1.91E-01	5DPI_iCoV2 + Alum
**linoleate 13S-lipoxygenase activity**	GO:0016165	4	2	0.04	+	48.88	1.49E-03	1.96E-01	5DPI_iCoV2 + Alum
**arachidonate 15-lipoxygenase activity**	GO:0050473	4	2	0.04	+	48.88	1.49E-03	2.01E-01	5DPI_iCoV2 + Alum
**metalloendopeptidase activity**	GO:0004222	118	6	1.21	+	4.97	1.67E-03	2.08E-01	5DPI_iCoV2 + Alum
**RNA nuclease activity**	GO:0004540	121	6	1.24	+	4.85	1.88E-03	2.29E-01	5DPI_iCoV2 + Alum
**CCR3 chemokine receptor binding**	GO:0031728	5	2	0.05	+	39.11	2.07E-03	2.46E-01	5DPI_iCoV2 + Alum
**ATP binding**	GO:0005524	1408	4	14.4	-	0.28	2.2E-03	2.55E-01	5DPI_iCoV2 + Alum
**ribonucleotide binding**	GO:0032553	1832	7	18.74	-	0.37	2.25E-03	2.56E-01	5DPI_iCoV2 + Alum

**protein binding**	GO:0005515	9462	120	96.78	+	1.24	2.27E-03	2.51E-01	5DPI_iCoV2 + Alum
**enzyme regulator activity**	GO:0030234	1285	25	13.14	+	1.9	2.4E-03	2.6E-01	5DPI_iCoV2 + Alum
**C-C chemokine receptor activity**	GO:0016493	24	3	0.25	+	12.22	2.5E-03	2.66E-01	5DPI_iCoV2 + Alum
**serine-type endopeptidase inhibitor activity**	GO:0004867	129	6	1.32	+	4.55	2.56E-03	2.66E-01	5DPI_iCoV2 + Alum
**chemokine receptor activity**	GO:0004950	25	3	0.26	+	11.73	2.78E-03	2.72E-01	5DPI_iCoV2 + Alum
**C-C chemokine binding**	GO:0019957	25	3	0.26	+	11.73	2.78E-03	2.78E-01	5DPI_iCoV2 + Alum
**G protein-coupled chemoattractant receptor activity**	GO:0001637	25	3	0.26	+	11.73	2.78E-03	2.84E-01	5DPI_iCoV2 + Alum
**heterocyclic compound binding**	GO:1901363	5371	36	54.94	-	0.66	2.91E-03	2.79E-01	5DPI_iCoV2 + Alum
**monosaccharide binding**	GO:0048029	92	5	0.94	+	5.31	3.08E-03	2.85E-01	5DPI_iCoV2 + Alum
**oxidoreductase activity, acting on single donors with incorporation of molecular oxygen, incorporation of two atoms of oxygen**	GO:0016702	26	3	0.27	+	11.28	3.08E-03	2.9E-01	5DPI_iCoV2 + Alum
**hydrolase activity**	GO:0016787	2363	39	24.17	+	1.61	3.29E-03	2.99E-01	5DPI_iCoV2 + Alum
**oxidoreductase activity, acting on single donors with incorporation of molecular oxygen**	GO:0016701	27	3	0.28	+	10.86	3.4E-03	3.03E-01	5DPI_iCoV2 + Alum
**cytoskeletal protein binding**	GO:0008092	1021	2	10.44	-	0.19	3.43E-03	3E-01	5DPI_iCoV2 + Alum
**CCR2 chemokine receptor binding**	GO:0031727	7	2	0.07	+	27.93	3.51E-03	3.02E-01	5DPI_iCoV2 + Alum
**enzyme inhibitor activity**	GO:0004857	410	11	4.19	+	2.62	3.92E-03	3.31E-01	5DPI_iCoV2 + Alum
**purine nucleotide binding**	GO:0017076	1924	8	19.68	-	0.41	3.97E-03	3.3E-01	5DPI_iCoV2 + Alum
**cytokine binding**	GO:0019955	142	6	1.45	+	4.13	4.03E-03	3.3E-01	5DPI_iCoV2 + Alum
**dicarboxylic acid transmembrane transporter activity**	GO:0005310	29	3	0.3	+	10.11	4.09E-03	3.29E-01	5DPI_iCoV2 + Alum
**adenyl ribonucleotide binding**	GO:0032559	1479	5	15.13	-	0.33	4.35E-03	3.44E-01	5DPI_iCoV2 + Alum
**laminin binding**	GO:0043236	30	3	0.31	+	9.78	4.47E-03	3.48E-01	5DPI_iCoV2 + Alum
**metallopeptidase activity**	GO:0008237	198	7	2.03	+	3.46	5E-03	3.84E-01	5DPI_iCoV2 + Alum
**integrin binding**	GO:0005178	152	6	1.55	+	3.86	5.53E-03	4.18E-01	5DPI_iCoV2 + Alum
**mannose binding**	GO:0005537	33	3	0.34	+	8.89	5.72E-03	4.26E-01	5DPI_iCoV2 + Alum
**chemokine binding**	GO:0019956	34	3	0.35	+	8.63	6.18E-03	4.53E-01	5DPI_iCoV2 + Alum
**solute:monoatomic cation symporter activity**	GO:0015294	113	5	1.16	+	4.33	7.06E-03	5.11E-01	5DPI_iCoV2 + Alum
**transferase activity**	GO:0016740	2239	11	22.9	-	0.48	7.24E-03	5.16E-01	5DPI_iCoV2 + Alum
**odorant binding**	GO:0005549	534	0	5.46	-	< 0.01	7.33E-03	5.15E-01	5DPI_iCoV2 + Alum

**transition metal ion binding**	GO:0046914	989	19	10.12	+	1.88	8.99E-03	6.23E-01	5DPI_iCoV2 + Alum
**iron ion binding**	GO:0005506	171	6	1.75	+	3.43	9.44E-03	6.46E-01	5DPI_iCoV2 + Alum
**poly-specific ribonuclease activity**	GO:0004535	13	2	0.13	+	15.04	9.83E-03	6.63E-01	5DPI_iCoV2 + Alum
**organic cyclic compound binding**	GO:0097159	5453	39	55.78	-	0.7	1.01E-02	6.75E-01	5DPI_iCoV2 + Alum
**water transmembrane transporter activity**	GO:0005372	14	2	0.14	+	13.97	1.12E-02	7.33E-01	5DPI_iCoV2 + Alum
**chemoattractant activity**	GO:0042056	43	3	0.44	+	6.82	1.13E-02	7.22E-01	5DPI_iCoV2 + Alum
**heparin binding**	GO:0008201	178	6	1.82	+	3.3	1.13E-02	7.32E-01	5DPI_iCoV2 + Alum
**C4-dicarboxylate transmembrane transporter activity**	GO:0015556	15	2	0.15	+	13.04	1.26E-02	7.74E-01	5DPI_iCoV2 + Alum
**phospholipase activator activity**	GO:0016004	15	2	0.15	+	13.04	1.26E-02	7.83E-01	5DPI_iCoV2 + Alum
**1-phosphatidylinositol-3-kinase regulator activity**	GO:0046935	15	2	0.15	+	13.04	1.26E-02	7.93E-01	5DPI_iCoV2 + Alum
**adenyl nucleotide binding**	GO:0030554	1588	7	16.24	-	0.43	1.32E-02	8.04E-01	5DPI_iCoV2 + Alum
**G protein-coupled amine receptor activity**	GO:0008227	46	3	0.47	+	6.38	1.34E-02	8.06E-01	5DPI_iCoV2 + Alum
**icosanoid receptor activity**	GO:0004953	16	2	0.16	+	12.22	1.4E-02	8.34E-01	5DPI_iCoV2 + Alum
**G protein-coupled receptor activity**	GO:0004930	743	15	7.6	+	1.97	1.41E-02	8.3E-01	5DPI_iCoV2 + Alum
**hormone activity**	GO:0005179	137	5	1.4	+	3.57	1.49E-02	8.65E-01	5DPI_iCoV2 + Alum
**insulin-like growth factor receptor binding**	GO:0005159	17	2	0.17	+	11.5	1.56E-02	8.94E-01	5DPI_iCoV2 + Alum
**molecular function inhibitor activity**	GO:0140678	552	12	5.65	+	2.13	1.57E-02	8.91E-01	5DPI_iCoV2 + Alum
**calcium ion binding**	GO:0005509	631	13	6.45	+	2.01	1.58E-02	8.85E-01	5DPI_iCoV2 + Alum
**lipase activator activity**	GO:0060229	19	2	0.19	+	10.29	1.89E-02	1E+00	5DPI_iCoV2 + Alum
**oligosaccharide binding**	GO:0070492	19	2	0.19	+	10.29	1.89E-02	1E+00	5DPI_iCoV2 + Alum
**nuclease activity**	GO:0004518	202	6	2.07	+	2.9	1.95E-02	1E+00	5DPI_iCoV2 + Alum
**nucleic acid binding**	GO:0003676	3371	22	34.48	-	0.64	1.96E-02	1E+00	5DPI_iCoV2 + Alum
**interleukin-13 receptor binding**	GO:0005144	1	1	0.01	+	97.76	2.01E-02	8.59E-01	5DPI_iCoV2 + Alum
**amidinotransferase activity**	GO:0015067	1	1	0.01	+	97.76	2.01E-02	8.67E-01	5DPI_iCoV2 + Alum
**glycine amidinotransferase activity**	GO:0015068	1	1	0.01	+	97.76	2.01E-02	8.74E-01	5DPI_iCoV2 + Alum
**hepoxilin-epoxide hydrolase activity**	GO:0047977	1	1	0.01	+	97.76	2.01E-02	8.82E-01	5DPI_iCoV2 + Alum
**amino-acid betaine transmembrane transporter activity**	GO:0015199	1	1	0.01	+	97.76	2.01E-02	8.9E-01	5DPI_iCoV2 + Alum
**NADPH phosphatase activity**	GO:0102757	1	1	0.01	+	97.76	2.01E-02	8.98E-01	5DPI_iCoV2 + Alum
**C5a anaphylatoxin chemotactic receptor binding**	GO:0031714	1	1	0.01	+	97.76	2.01E-02	9.06E-01	5DPI_iCoV2 + Alum

**C5L2 anaphylatoxin chemotactic receptor binding**	GO:0031715	1	1	0.01	+	97.76	2.01E-02	9.14E-01	5DPI_iCoV2 + Alum
**CCR4 chemokine receptor binding**	GO:0031729	1	1	0.01	+	97.76	2.01E-02	9.23E-01	5DPI_iCoV2 + Alum
**galactose:sodium symporter activity**	GO:0015371	1	1	0.01	+	97.76	2.01E-02	9.31E-01	5DPI_iCoV2 + Alum
**high-affinity IgE receptor activity**	GO:0019768	1	1	0.01	+	97.76	2.01E-02	9.4E-01	5DPI_iCoV2 + Alum
**acetylgalactosaminyl-O-glycosyl-glycoprotein beta-1,6-N-acetylglucosaminyltransferase activity**	GO:0047225	1	1	0.01	+	97.76	2.01E-02	9.49E-01	5DPI_iCoV2 + Alum
**acetylgalactosaminyl-O-glycosyl-seryl-glycoprotein beta-1,6-N-acetylglucosaminyltransferase activity**	GO:0106325	1	1	0.01	+	97.76	2.01E-02	9.58E-01	5DPI_iCoV2 + Alum
**acetylgalactosaminyl-O-glycosyl-threonyl-glycoprotein beta-1,6-N-acetylglucosaminyltransferase activity**	GO:0106326	1	1	0.01	+	97.76	2.01E-02	9.67E-01	5DPI_iCoV2 + Alum
**cholesterol 25-hydroxylase activity**	GO:0001567	1	1	0.01	+	97.76	2.01E-02	9.76E-01	5DPI_iCoV2 + Alum
**all-trans retinoic acid 18-hydroxylase activity**	GO:0062183	1	1	0.01	+	97.76	2.01E-02	9.86E-01	5DPI_iCoV2 + Alum
**interleukin-33 receptor binding**	GO:0002112	1	1	0.01	+	97.76	2.01E-02	9.96E-01	5DPI_iCoV2 + Alum
**propane-1,3-diamine oxidase activity**	GO:0052600	1	1	0.01	+	97.76	2.01E-02	1E+00	5DPI_iCoV2 + Alum
**aconitate decarboxylase activity**	GO:0047613	1	1	0.01	+	97.76	2.01E-02	1E+00	5DPI_iCoV2 + Alum
**NADP phosphatase activity**	GO:0019178	1	1	0.01	+	97.76	2.01E-02	1E+00	5DPI_iCoV2 + Alum
**methylputrescine oxidase activity**	GO:0052599	1	1	0.01	+	97.76	2.01E-02	1E+00	5DPI_iCoV2 + Alum
**histamine oxidase activity**	GO:0052598	1	1	0.01	+	97.76	2.01E-02	1E+00	5DPI_iCoV2 + Alum
**G protein-coupled bile acid receptor activity**	GO:0038182	1	1	0.01	+	97.76	2.01E-02	1E+00	5DPI_iCoV2 + Alum
**interleukin-33 binding**	GO:0002113	1	1	0.01	+	97.76	2.01E-02	1E+00	5DPI_iCoV2 + Alum
**salt transmembrane transporter activity**	GO:1901702	582	12	5.95	+	2.02	2.05E-02	8.69E-01	5DPI_iCoV2 + Alum
**phosphatidylinositol 3-kinase regulator activity**	GO:0035014	20	2	0.2	+	9.78	2.06E-02	8.66E-01	5DPI_iCoV2 + Alum
**symporter activity**	GO:0015293	150	5	1.53	+	3.26	2.09E-02	8.71E-01	5DPI_iCoV2 + Alum
**growth factor receptor binding**	GO:0070851	151	5	1.54	+	3.24	2.15E-02	8.78E-01	5DPI_iCoV2 + Alum
**monooxygenase activity**	GO:0004497	151	5	1.54	+	3.24	2.15E-02	8.86E-01	5DPI_iCoV2 + Alum
**G protein-coupled serotonin receptor activity**	GO:0004993	22	2	0.23	+	8.89	2.43E-02	9.87E-01	5DPI_iCoV2 + Alum
**carboxylic acid binding**	GO:0031406	217	6	2.22	+	2.7	2.64E-02	1E+00	5DPI_iCoV2 + Alum
**heme binding**	GO:0020037	160	5	1.64	+	3.06	2.65E-02	1E+00	5DPI_iCoV2 + Alum
**serotonin receptor activity**	GO:0099589	24	2	0.25	+	8.15	2.83E-02	1E+00	5DPI_iCoV2 + Alum

**granulocyte colony-stimulating factor receptor binding**	GO:0005130	2	1	0.02	+	48.88	3.01E-02	1E+00	5DPI_iCoV2 + Alum
**1-propan-2-amine binding**	GO:0071886	2	1	0.02	+	48.88	3.01E-02	1E+00	5DPI_iCoV2 + Alum
**prostacyclin receptor activity**	GO:0016501	2	1	0.02	+	48.88	3.01E-02	1E+00	5DPI_iCoV2 + Alum
**activin receptor antagonist activity**	GO:0038102	2	1	0.02	+	48.88	3.01E-02	1E+00	5DPI_iCoV2 + Alum
**folic acid receptor activity**	GO:0061714	2	1	0.02	+	48.88	3.01E-02	1E+00	5DPI_iCoV2 + Alum
**interleukin-33 receptor activity**	GO:0002114	2	1	0.02	+	48.88	3.01E-02	1E+00	5DPI_iCoV2 + Alum
**leukotriene B4 receptor activity**	GO:0001632	2	1	0.02	+	48.88	3.01E-02	1E+00	5DPI_iCoV2 + Alum
**inorganic diphosphate transmembrane transporter activity**	GO:0030504	2	1	0.02	+	48.88	3.01E-02	1E+00	5DPI_iCoV2 + Alum
**long-chain-alcohol O-fatty-acyltransferase activity**	GO:0047196	2	1	0.02	+	48.88	3.01E-02	1E+00	5DPI_iCoV2 + Alum
**arginase activity**	GO:0004053	2	1	0.02	+	48.88	3.01E-02	1E+00	5DPI_iCoV2 + Alum
**cholesterol monooxygenase activity**	GO:0008386	2	1	0.02	+	48.88	3.01E-02	1E+00	5DPI_iCoV2 + Alum
**arachidoyl-CoA:1-dodecanol O-acyltransferase activity**	GO:0102966	2	1	0.02	+	48.88	3.01E-02	1E+00	5DPI_iCoV2 + Alum
**fructose 1,6-bisphosphate 1-phosphatase activity**	GO:0042132	2	1	0.02	+	48.88	3.01E-02	1E+00	5DPI_iCoV2 + Alum
**phosphatidylinositol-3,4,5-trisphosphate 5-phosphatase activity**	GO:0034485	2	1	0.02	+	48.88	3.01E-02	1E+00	5DPI_iCoV2 + Alum
**N-acetyllactosaminide beta-1,6-N-acetylglucosaminyltransferase activity**	GO:0008109	2	1	0.02	+	48.88	3.01E-02	1E+00	5DPI_iCoV2 + Alum
**alpha-2C adrenergic receptor binding**	GO:0031696	2	1	0.02	+	48.88	3.01E-02	1E+00	5DPI_iCoV2 + Alum
**alpha-glucoside transmembrane transporter activity**	GO:0015151	2	1	0.02	+	48.88	3.01E-02	1E+00	5DPI_iCoV2 + Alum
**hepoxilin A3 synthase activity**	GO:0051120	2	1	0.02	+	48.88	3.01E-02	1E+00	5DPI_iCoV2 + Alum
**lactoperoxidase activity**	GO:0140825	2	1	0.02	+	48.88	3.01E-02	1E+00	5DPI_iCoV2 + Alum
**oncostatin-M receptor binding**	GO:0005147	2	1	0.02	+	48.88	3.01E-02	1E+00	5DPI_iCoV2 + Alum
**interleukin-5 receptor binding**	GO:0005137	2	1	0.02	+	48.88	3.01E-02	1E+00	5DPI_iCoV2 + Alum
**tetrapyrrole binding**	GO:0046906	169	5	1.73	+	2.89	3.23E-02	1E+00	5DPI_iCoV2 + Alum
**peptidase activator activity**	GO:0016504	66	3	0.68	+	4.44	3.29E-02	1E+00	5DPI_iCoV2 + Alum
**active monoatomic ion transmembrane transporter activity**	GO:0022853	229	6	2.34	+	2.56	3.29E-02	1E+00	5DPI_iCoV2 + Alum
**oxidoreductase activity, acting on paired donors, with incorporation or reduction of molecular oxygen**	GO:0016705	229	6	2.34	+	2.56	3.29E-02	1E+00	5DPI_iCoV2 + Alum
**carboxylic acid transmembrane transporter activity**	GO:0046943	170	5	1.74	+	2.88	3.3E-02	1E+00	5DPI_iCoV2 + Alum

**organic acid transmembrane transporter activity**	GO:0005342	171	5	1.75	+	2.86	3.37E-02	1E+00	5DPI_iCoV2 + Alum
**organic acid binding**	GO:0043177	233	6	2.38	+	2.52	3.52E-02	1E+00	5DPI_iCoV2 + Alum
**sulfur compound binding**	GO:1901681	297	7	3.04	+	2.3	3.57E-02	1E+00	5DPI_iCoV2 + Alum
**tubulin binding**	GO:0015631	385	0	3.94	-	< 0.01	3.62E-02	1E+00	5DPI_iCoV2 + Alum
**bicarbonate transmembrane transporter activity**	GO:0015106	28	2	0.29	+	6.98	3.69E-02	1E+00	5DPI_iCoV2 + Alum
**L-amino acid transmembrane transporter activity**	GO:0015179	70	3	0.72	+	4.19	3.8E-02	1E+00	5DPI_iCoV2 + Alum
**amino acid:monoatomic cation symporter activity**	GO:0005416	29	2	0.3	+	6.74	3.92E-02	1E+00	5DPI_iCoV2 + Alum
**glycosaminoglycan binding**	GO:0005539	240	6	2.45	+	2.44	3.96E-02	1E+00	5DPI_iCoV2 + Alum
**chondroitin 4-sulfotransferase activity**	GO:0047756	3	1	0.03	+	32.59	3.99E-02	1E+00	5DPI_iCoV2 + Alum
**C-4 methylsterol oxidase activity**	GO:0000254	3	1	0.03	+	32.59	3.99E-02	1E+00	5DPI_iCoV2 + Alum
**Toll-like receptor 4 binding**	GO:0035662	3	1	0.03	+	32.59	3.99E-02	1E+00	5DPI_iCoV2 + Alum
**nitric-oxide synthase activity**	GO:0004517	3	1	0.03	+	32.59	3.99E-02	1E+00	5DPI_iCoV2 + Alum
**dopamine beta-monooxygenase activity**	GO:0004500	3	1	0.03	+	32.59	3.99E-02	1E+00	5DPI_iCoV2 + Alum
**bile acid receptor activity**	GO:0038181	3	1	0.03	+	32.59	3.99E-02	1E+00	5DPI_iCoV2 + Alum
**beta-1,3-galactosyl-O-glycosyl-glycoprotein beta-1,6-N-acetylglucosaminyltransferase activity**	GO:0003829	3	1	0.03	+	32.59	3.99E-02	1E+00	5DPI_iCoV2 + Alum
**Gq/11-coupled serotonin receptor activity**	GO:0001587	3	1	0.03	+	32.59	3.99E-02	1E+00	5DPI_iCoV2 + Alum
**tetrahydrobiopterin binding**	GO:0034617	3	1	0.03	+	32.59	3.99E-02	1E+00	5DPI_iCoV2 + Alum
**type II activin receptor binding**	GO:0070699	3	1	0.03	+	32.59	3.99E-02	1E+00	5DPI_iCoV2 + Alum
**alpha2-adrenergic receptor activity**	GO:0004938	3	1	0.03	+	32.59	3.99E-02	1E+00	5DPI_iCoV2 + Alum
**iodide transmembrane transporter activity**	GO:0015111	3	1	0.03	+	32.59	3.99E-02	1E+00	5DPI_iCoV2 + Alum
**tryptophan 2,3-dioxygenase activity**	GO:0004833	3	1	0.03	+	32.59	3.99E-02	1E+00	5DPI_iCoV2 + Alum
**sodium:dicarboxylate symporter activity**	GO:0017153	3	1	0.03	+	32.59	3.99E-02	1E+00	5DPI_iCoV2 + Alum
**glucoside transmembrane transporter activity**	GO:0042947	3	1	0.03	+	32.59	3.99E-02	1E+00	5DPI_iCoV2 + Alum
**interleukin-4 receptor binding**	GO:0005136	3	1	0.03	+	32.59	3.99E-02	1E+00	5DPI_iCoV2 + Alum
**cell adhesion molecule binding**	GO:0050839	305	7	3.12	+	2.24	4.02E-02	1E+00	5DPI_iCoV2 + Alum
**3'-5'-RNA exonuclease activity**	GO:0000175	30	2	0.31	+	6.52	4.15E-02	1E+00	5DPI_iCoV2 + Alum
**organic anion transmembrane transporter activity**	GO:0008514	243	6	2.49	+	2.41	4.16E-02	1E+00	5DPI_iCoV2 + Alum
**inorganic molecular entity transmembrane transporter activity**	GO:0015318	651	12	6.66	+	1.8	4.66E-02	1E+00	5DPI_iCoV2 + Alum

**RNA exonuclease activity, producing 5'-phosphomonoesters**	GO:0016896	33	2	0.34	+	5.93	4.88E-02	1E+00	5DPI_iCoV2 + Alum
**protein-RNA adaptor activity**	GO:0140517	4	1	0.04	+	24.44	4.96E-02	1E+00	5DPI_iCoV2 + Alum
**diamine oxidase activity**	GO:0052597	4	1	0.04	+	24.44	4.96E-02	1E+00	5DPI_iCoV2 + Alum
**L-lactate dehydrogenase activity**	GO:0004459	4	1	0.04	+	24.44	4.96E-02	1E+00	5DPI_iCoV2 + Alum
**palmitoyl-CoA 9-desaturase activity**	GO:0032896	4	1	0.04	+	24.44	4.96E-02	1E+00	5DPI_iCoV2 + Alum
**N-acetyl-beta-D-galactosaminidase activity**	GO:0102148	4	1	0.04	+	24.44	4.96E-02	1E+00	5DPI_iCoV2 + Alum
**amylin receptor activity**	GO:0097643	4	1	0.04	+	24.44	4.96E-02	1E+00	5DPI_iCoV2 + Alum
**group III metabotropic glutamate receptor activity**	GO:0001642	4	1	0.04	+	24.44	4.96E-02	1E+00	5DPI_iCoV2 + Alum
**adrenomedullin receptor activity**	GO:0001605	4	1	0.04	+	24.44	4.96E-02	1E+00	5DPI_iCoV2 + Alum
**histone acetyltransferase regulator activity**	GO:0035034	4	1	0.04	+	24.44	4.96E-02	1E+00	5DPI_iCoV2 + Alum
**norepinephrine binding**	GO:0051380	4	1	0.04	+	24.44	4.96E-02	1E+00	5DPI_iCoV2 + Alum
**urea channel activity**	GO:0015265	4	1	0.04	+	24.44	4.96E-02	1E+00	5DPI_iCoV2 + Alum
**glycerol channel activity**	GO:0015254	4	1	0.04	+	24.44	4.96E-02	1E+00	5DPI_iCoV2 + Alum
**leukotriene receptor activity**	GO:0004974	4	1	0.04	+	24.44	4.96E-02	1E+00	5DPI_iCoV2 + Alum
**tricarboxylic acid transmembrane transporter activity**	GO:0015142	4	1	0.04	+	24.44	4.96E-02	1E+00	5DPI_iCoV2 + Alum
**citrate transmembrane transporter activity**	GO:0015137	4	1	0.04	+	24.44	4.96E-02	1E+00	5DPI_iCoV2 + Alum
**oxidoreductase activity, acting on paired donors, with incorporation or reduction of molecular oxygen, reduced ascorbate as one donor, and incorporation of one atom of oxygen**	GO:0016715	4	1	0.04	+	24.44	4.96E-02	1E+00	5DPI_iCoV2 + Alum
