# Supplementary material for: Adjuvant-dependent impact of inactivated SARS-CoV-2 vaccines during heterologous infection by a SARS-related coronavirus
Source: Nat Commun. 2024 May 3;15:3738. doi: 10.1038/s41467-024-47450-x (PMC11068739; doi:10.1038/s41467-024-47450-x)
Supplement: Supplementary file 3 — Description of Additional Supplementary Files [file 41467_2024_47450_MOESM3_ESM.pdf]

File Name: Supplementary Data 1

Description: Supplementary information related to primer-probe sets used for quantitative RT-PCR. Information provided for each primer-probe set includes commercial vendor, gene, NCBI Reference Sequence, probe, catalogue number, exon location, and sequence. All primer-probe sequences besides those used for *Gapdh* (which are proprietary per the commercial vendor) are provided.
