# Supplementary material for: Adjuvant-dependent impact of inactivated SARS-CoV-2 vaccines during heterologous infection by a SARS-related coronavirus
Source: Nat Commun. 2024 May 3;15:3738. doi: 10.1038/s41467-024-47450-x (PMC11068739; doi:10.1038/s41467-024-47450-x)
Supplement: Supplementary file 5 — Reporting Summary [file 41467_2024_47450_MOESM5_ESM.pdf]

nature portfolio

Corresponding author(s): Mark Heise

Last updated by author(s): Feb 24, 2024

Reporting Summary

Nature Portfolio wishes to improve the reproducibility of the work that we publish. This form provides structure for consistency and transparency in reporting. For further information on Nature Portfolio policies, see our Editorial Policies and the Editorial Policy Checklist.

Statistics

For all statistical analyses, confirm that the following items are present in the figure legend, table legend, main text, or Methods section.

n/a	Confirmed	
☐	☒	The exact sample size (*n*) for each experimental group/condition, given as a discrete number and unit of measurement
☐	☒	A statement on whether measurements were taken from distinct samples or whether the same sample was measured repeatedly
☐	☒	The statistical test(s) used AND whether they are one- or two-sided<br>*Only common tests should be described solely by name; describe more complex techniques in the Methods section.*
☒	☐	A description of all covariates tested
☐	☒	A description of any assumptions or corrections, such as tests of normality and adjustment for multiple comparisons
☐	☒	A full description of the statistical parameters including central tendency (e.g. means) or other basic estimates (e.g. regression coefficient) AND variation (e.g. standard deviation) or associated estimates of uncertainty (e.g. confidence intervals)
☐	☒	For null hypothesis testing, the test statistic (e.g. *F*, *t*, *r*) with confidence intervals, effect sizes, degrees of freedom and *P* value noted<br>*Give P values as exact values whenever suitable.*
☒	☐	For Bayesian analysis, information on the choice of priors and Markov chain Monte Carlo settings
☒	☐	For hierarchical and complex designs, identification of the appropriate level for tests and full reporting of outcomes
☒	☐	Estimates of effect sizes (e.g. Cohen's *d*, Pearson's *r*), indicating how they were calculated

Our web collection on statistics for biologists contains articles on many of the points above.

Software and code

Policy information about availability of computer code

Data collection	No software was used for data collection in this study.
Data analysis	Statistical analyses were performed using Graphpad Prism 9 and 10 (except for RNA-Seq analysis).<br><br>RNA sequencing analysis used publicly available software as follows. FastQC (v0.11.9) was used to confirm data quality. Salmon (v1.10.0) was used to quantify transcripts. tximport (v1.28.0) was used to import gene-level count matrices for use in differential expression analysis. DESeq1 (v1.40.2) was used to normalize count data. PANTER 17.0 was used for Gene Ontology (GO) analyses. All analyses were run in Bioconductor and the R statistical package.<br><br>These analysis methods are also described in the Methods section.

For manuscripts utilizing custom algorithms or software that are central to the research but not yet described in published literature, software must be made available to editors and reviewers. We strongly encourage code deposition in a community repository (e.g. GitHub). See the Nature Portfolio guidelines for submitting code & software for further information.

Data

Policy information about availability of data

All manuscripts must include a data availability statement. This statement should provide the following information, where applicable:

- Accession codes, unique identifiers, or web links for publicly available datasets
- A description of any restrictions on data availability
- For clinical datasets or third party data, please ensure that the statement adheres to our policy

Source data are submitted to Figshare (DOI pending).

RNA Sequencing raw .fastq data files are submitted to the Sequence Read Archive (SRA) database under BioProject ID PRJNA1022427. Raw source data are provided with this publication.

Research involving human participants, their data, or biological material

Policy information about studies with human participants or human data. See also policy information about sex, gender (identity/presentation), and sexual orientation and race, ethnicity and racism.

Reporting on sex and gender	This study did not involve human participants, their data, or biological material. Therefore, this information has not been collected.
Reporting on race, ethnicity, or other socially relevant groupings	This study did not involve human participants, their data, or biological material. Therefore, this information has not been collected.
Population characteristics	This study did not involve human participants, their data, or biological material. Therefore, this information has not been collected.
Recruitment	This study did not involve human participants, their data, or biological material. Therefore, no human participants were recruited.
Ethics oversight	This study did not involve human participants, their data, or biological material. Therefore, no ethics oversight for research involving human participants was required.

Note that full information on the approval of the study protocol must also be provided in the manuscript.

Field-specific reporting

Please select the one below that is the best fit for your research. If you are not sure, read the appropriate sections before making your selection.

☒ Life sciences ☐ Behavioural & social sciences ☐ Ecological, evolutionary & environmental sciences

For a reference copy of the document with all sections, see nature.com/documents/nr-reporting-summary-flat.pdf

Life sciences study design

All studies must disclose on these points even when the disclosure is negative.

Sample size	Power calculations based on expected magnitude and variability of effect sizes were used to calculate mouse sample sizes. Because multiple parameters were measured to evaluate experimental outcomes, power calculations were based on the parameter expected to exhibit the greatest variability. Sample sizes based on power calculations were generally increased to account for mouse attrition throughout the experiment.
Data exclusions	For quantitative immunohistochemistry analysis, to increase the specificity of detection for the desired cell types (EPX+ eosinophils or CD4+ T helper cells), low-intensity signals were excluded from analysis. These exclusion criteria were pre-established and were applied equally to all groups and samples.<br><br>No other data were excluded from analysis.
Replication	Some experiments were not replicated because (1) length of some experiments prohibited timely replication and (2) results from unreplicated experiments involved high magnitude effects and were consistent with replicated findings observed throughout the study.<br><br>Experiments that were replicated (numbers of replications in parentheses): Fig. 1a (4), Fig. 1d (3), Fig. 1e (2), Fig. 1f (2), Fig. 1h (2), Fig. 2a (4), Fig. 2b (2), Fig. 2d (3), Fig. 2e (2), Fig. 2g (2), Fig. 5a (2), Fig. 5c (2), Fig. 5d (2), Fig. 5e (2), Fig. 6a (2), Extended Data Fig. 2a-j (2), Extended Data Fig. 3a (2), Extended Data Fig. 4a (3), Extended Data Fig. 4b (3).
Randomization	Mice were randomly allocated into different experimental groups.

Blinding

Investigators were blinded during collection/analysis of all experimental parameters besides clinical scoring of Rs-SHC014-CoV (SHC014)-infected animals and whole body plethsymography.

Blinding during clinical scoring of SHC014-infected animals was not deemed to be central to the study because the SHC014 challenge model does not involve severe clinical disease. The SHC014 challenge model used is an established model and is known to not cause overt severe clinical disease in mice. Therefore, the experiments were designed to analyze other outcomes of infection, including pulmonary pathology including inflammatory infiltrates, pulmonary viral load, and pulmonary function. Unblinded clinical scoring results shown in Extended Data Fig. 4 confirmed previous findings by others (Menachery et al, Nature Medicine, 2015) that SHC014-infected mice exhibit very low levels of clinical disease.

Blinding during whole body plethysmography was not possible because mouse identification was required during data acquisition. Whole body plethysmography is also an objective quantitative method that is unlikely to be affected by unblinded investigators.

Reporting for specific materials, systems and methods

We require information from authors about some types of materials, experimental systems and methods used in many studies. Here, indicate whether each material, system or method listed is relevant to your study. If you are not sure if a list item applies to your research, read the appropriate section before selecting a response.

Materials & experimental systems

n/a	Involved in the study
☐	☒ Antibodies
☐	☒ Eukaryotic cell lines
☒	☐ Palaeontology and archaeology
☐	☒ Animals and other organisms
☒	☐ Clinical data
☐	☒ Dual use research of concern
☒	☐ Plants

Methods

n/a	Involved in the study
☒	☐ ChIP-seq
☒	☐ Flow cytometry
☒	☐ MRI-based neuroimaging

Antibodies

Antibodies used

- Anti-EPX polyclonal antibody: Invitrogen, CAT # PA5-62200, LOT # YC3868704
- Anti-CD4 monoclonal antibody: Abcam, CAT # ab183685, Clone EPR19514, LOT # GR3240246-13
- Anti-SARS polyclonal antibody : Novus Biological, CAT # NB100-56576, LOT # 111003c-07
- C3 compliment antiserum: MP Biomedical, CAT # 55444, LOT # 08296
- Anti-Arginase monoclonal antibody: Cell Signaling Technology, CAT # 93668S, Clone D4E3M, LOT # 4
- Rat IgG2b isotype control monoclonal antibody: BioXCell, CAT # BE0090, Clone LTF-2, LOT # 831022J3
- Anti-CD4 monoclonal antibody: BioXCell, CAT # BE0003-1, Clone GK1.5, LOT # 805422S1
- Discovery OmniMap polyclonal anti-rabbit HRP: Roche, CAT # 760-4311, LOT # K23370
- Polyclonal anti-goat HRP: Dako, CAT # P0160, LOT # 20066257
- Novolink Polymer polyclonal anti-rabbit HRP: Leica, CAT # RE7260-K, LOT # 6108804

Primary and secondary antibody dilution information is described in the Methods.

Validation

- Anti-EPX polyclonal antibody: Invitrogen, CAT # PA5-62200, LOT # YC3868704
o Validation: The website of Invitrogen (commercial provider) states that all of their antibodies come with a performance guarantee. The product page has additional details, a performance guarantee label, and a cited publication demonstrating use of the antibody, as well as a technical data sheet describing validation techniques such as purification via antigen affinity chromatography and demonstrated use in immunohistochemistry (https://www.thermofisher.com/antibody/product/EPX-Antibody-Polyclonal/PA5-62200).

- Anti-CD4 monoclonal antibody: Abcam, CAT # ab183685, LOT # GR3240246-13
o Validation: The website of Abcam (commercial provider) states that they guarantee the quality of their products including antibodies. The product page has additional details, numerous cited publications demonstrating use of the antibody, as well as a technical data sheet describing validation techniques such as purification via Protein A, antigen-binding verification via western blot, and demonstrated use in immunohistochemistry (https://www.abcam.com/products/primary-antibodies/cd4-antibody-epr19514-ab183685.html?productWallTab=ShowAll).

- Anti-SARS polyclonal antibody : Novus Biological, CAT # NB100-56576, LOT # 111003c-07
o Validation: The website of Novus Biologicals (commercial provider) states that they guarantee the quality of their products including antibodies and that they validate their antibodies in accordance with the recommendations of the International Working Group for Antibody Validation (IWGAV). The product page has additional details, numerous cited publications demonstrating use of the antibody as well as a technical data sheet describing validation techniques such as purification via antigen affinity purification, antigen-binding verification via western blot, and demonstrated use in immunohistochemistry as well as cross-reactivity with SARS-CoV-2 but not MERS-CoV or human coronavirus HCoV-229E (https://www.novusbio.com/products/sars-nucleocapsid-protein-antibody_nb100-56576). Additionally, Menachery et al (A SARS-like cluster of circulating bat coronaviruses shows potential for human emergence. 2015. Nat Med 21:1508-13) demonstrate cross-reactivity with Rs-SCH014-CoV (SHC014) using

immunohistochemistry.

- C3 compliment antiserum: MP Biomedical, CAT # 55444, LOT # 08296
o Validation: The website of MP Biomedical (commercial provider) provides details regarding purification and specificity of the antigen, numerous cited publications demonstrating use of the antibody, and application notes confirming suitability for use in immunohistochemistry, as well as a technical data sheet describing validation techniques including antiserum purification via solid-phase absorption and antigen-binding verification via immunoelectrophoresis (https://www.mpbio.com/us/0855444-goat-antiserum-to-mouse-complement-c3).

- Anti-Arginase monoclonal antibody: Cell Signaling Technology, CA T# 93668S, LOT # 4
o Validation: The website Cell Signaling Technologies (commercial provider) states that all of their antibodies come with a performance guarantee. The product page has additional details and numerous cited publications demonstrating use of the antibody, as well as a technical data sheet describing validation techniques such as antigen-binding verification via western blot and demonstrated use in immunohistochemistry (https://www.cellsignal.com/products/primary-antibodies/arginase-1-d4e3m-xp-rabbit-mab/93668).

- Rat IgG2b isotype control monoclonal antibody: BioXCell, CAT # BE0090, LOT # 831022J3
o Validation: The website of BioXCell (commercial provider) states that all antibodies in their library have been validated for purity and quality. The product page has additional details and numerous cited publications demonstrating use of the antibody, as well as a technical data sheet describing validation techniques such as purification via SDS-PAGE and antigen-binding verification via western blotting (https://bioxcell.com/invivomab-rat-igg2b-isotype-control-anti-keyhole-limpet-hemocyanin-be0090).

- Anti-CD4 monoclonal antibody: BioXCell, CAT # BE0003-1, LOT # 805422S1
o Validation: The website of BioXCell (commercial provider) states that all antibodies in their library have been validated for purity and quality. The product page has additional details and numerous cited publications demonstrating use of the antibody, as well as a technical data sheet describing validation techniques such as purification via SDS-PAGE and antigen-binding verification via western blotting (https://bioxcell.com/invivomab-anti-mouse-cd4-be0003-1#tab_references).

Eukaryotic cell lines

Policy information about cell lines and Sex and Gender in Research

Cell line source(s)	Vero E6 USAMRIID cells were obtained from the laboratory of Ralph Baric. Vero E6 C1008 cells were obtained from ATCC (CRL-1586) through Operation Warp Speed (OWS).
Authentication	The cell lines used were not authenticated by our laboratory. The Vero E6 C1008 cells obtained from ATCC (CRL-1586) through Operation Warp Speed were authenticated by ATCC but we cannot locate the certificate of analysis for the lot number used.
Mycoplasma contamination	The Vero E6 C1008 cells obtained from ATCC (CRL-1586) were tested for mycoplasma contamination by ATCC but were not subsequently tested by our laboratory. The Vero E6 USAMRIID cells were not tested for mycoplasma contamination.
Commonly misidentified lines (See ICLAC register)	No commonly misidentified cell lines were used.

Animals and other research organisms

Policy information about studies involving animals; ARRIVE guidelines recommended for reporting animal research, and Sex and Gender in Research

Laboratory animals	BALB/cAnNHsd mice (Mus musculus) were purchased from Envigo/Inotiv (Stock 047) and were 6-8 weeks of age at first vaccination. a. Mice were housed in our ABSL3 facility on a 12:12 light cycle, kept within a temperature range of 20-23.3 °C and humidity range between 30-70%. Autoclaved cages (Tecniplast, EM500) were used with irradiated Bed-o-Cob (ScottPharma, Bed-o-Cob 4RB), ad libitum-irradiated chow (LabDiet, PicoLab Select Rodent 50 IF/6F 5V5R) and autoclaved water bottles. Cages were changed at least every 14 days and water bottles were changed every 7 days.
Wild animals	The study did not involve wild animals.
Reporting on sex	Only female mice were used due to the difficulty of maintaining male health related to aggressive behavior in a co-housing context. Use of male muse would have required a significant amount of single animal housing to limit fighting, and given the length of experiments and sample size requirements, use of female mice was optimal in terms of financial cost and space requirements.
Field-collected samples	The study did not involve samples collected from the field.
Ethics oversight	All studies were conducted under an animal use protocol approved by the Institutional Animal Care and Use Committee (IACUC) at the University of North Carolina at Chapel Hill, an AAALACi-certified institution, in accordance with the recommendations set forth by the 8th edition of the Guide for the Care and Use of Laboratory Animals.

Note that full information on the approval of the study protocol must also be provided in the manuscript.

Dual use research of concern

Policy information about dual use research of concern

Hazards

Could the accidental, deliberate or reckless misuse of agents or technologies generated in the work, or the application of information presented in the manuscript, pose a threat to:

No	Yes	
☐	☒	Public health
☐	☒	National security
☒	☐	Crops and/or livestock
☒	☐	Ecosystems
☒	☐	Any other significant area

Hazards

This study involved use of infectious clones of SARS-CoV-2 and SARS-related coronaviruses. Infectious clone (ic)SARS-CoV-2 wild-type derived from the D614 strain based on the WA1 sequence was used for vaccine production. Mouse-adapted SARS-CoV-2 MA10, mouse-adapted MA10 expressing SARS-CoV-2 B.1.351 spike, and Rs-SHC014-CoV (SHC014) were used for animal challenge experiments. Additionally, reporter viruses expressing nanoLuciferase (nLuc) (SARS-CoV-2-nLuc, B.1.351-nLuc, and SHC014-nLuc) were used for neutralization assays.

For examples of agents subject to oversight, see the United States Government Policy for Institutional Oversight of Life Sciences Dual Use Research of Concern.

Experiments of concern

Does the work involve any of these experiments of concern:

No	Yes	
☒	☐	Demonstrate how to render a vaccine ineffective
☒	☐	Confer resistance to therapeutically useful antibiotics or antiviral agents
☒	☐	Enhance the virulence of a pathogen or render a nonpathogen virulent
☒	☐	Increase transmissibility of a pathogen
☒	☐	Alter the host range of a pathogen
☒	☐	Enable evasion of diagnostic/detection modalities
☒	☐	Enable the weaponization of a biological agent or toxin
☒	☐	Any other potentially harmful combination of experiments and agents

Precautions and benefits

Biosecurity precautions

All activities involving coronaviruses were performed in an approved and registered biosafety level 3 (BSL-3/ABSL-3) facility following Standard Operating Procedures (SOPs) by trained personnel wearing appropriate personal protective equipment (PPE), including Powered Air Purifying Respirators (PAPRs), in accordance with the guidelines outlined in the CDC/NIH Biosafety in 'Microbiological and Biomedical Laboratories' (BMBL, 6th edition), as well as the NIH Guidelines for Research Involving Recombinant or Synthetic Nucleic Acid Molecules (April 2016). Additionally, Techniplast Sealsafe(TM) HEPA-filtered rodent housing was used for experiments involving viral infection of mice.

UNC BSL-3/ABSL-3 facilities are equipped with biosafety cabinets, incubators, centrifuges with containment features, cold storage units, an autoclave, sink, eyewash and life safety equipment, and mechanical system monitors and alarms to support effective isolation and containment of operations involving biohazardous agents. The anterooms to the BSL-3 facilities house Powered Air-Purifying Respirator (PAPR) charging stations, laboratory and safety supplies, and a changing area. Access to biohazardous agents is restricted by an entryway requiring a combination of swipe card and punch code for entry.

BSL-3/ABSL-3 facilities are under negative pressure, with redundant systems to ensure that negative pressure is maintained. All BSL-3 facilities have autoclaves to decontaminate waste materials as well as approved protocols for treatment or inactivation of all materials exiting the facilities. All personnel are extensively trained in basic virology and safety protocols before being approved for biohazardous agent work and undergo additional extensive training to work with BSL-3 pathogens including coronaviruses. Annual testing is performed to verify that biosafety cabinets, laboratory supply/exhaust systems (including alarms), and other laboratory equipment are functioning as designed. The BSL-3 facilities are secured at all times, and only personnel who have successfully completed extensive laboratory-specific training requirements are permitted to enter without an escort. All procedures involving coronaviruses use safety tested protocols to demonstrate virus inactivation prior to removal from BSL-3 facilities.

Biosecurity oversight

Standard Operating Procedures (SOPs) related to activities in BSL-3 facilities have been reviewed and approved by the UNC Institutional Biosafety Committee (IBC) and undergo both annual review and approval as well as updates as laboratory processes change or biosafety procedures evolve.

Benefits

SARS-related coronaviruses (SARS-r-CoVs) continue to represent a major pandemic threat. Because billions of people have already

Benefits	been vaccinated against SARS-CoV-2, we can prepare for future SARS-r-CoV pandemics by understanding how immunity from SARS-CoV-2 vaccines affects related coronaviruses that have the potential to spread to people. In this study, we investigated how SARS-CoV-2 vaccines impact SARS-r-CoV infections, which can inform the development of safe vaccines that can protect people from both SARS-CoV-2 and potential future coronaviruses.
Communication benefits	The benefits of communicating the findings of this investigation outweigh the associated risks. Information obtained from this study can be used to mitigate threats to public health posed by future novel coronavirus epidemics. All research activities were conducted in accordance with safety and containment practices outlined above. Moreover, no gain of function experiments were involved in this study.

Plants

Seed stocks	Plants/plant materials were not involved in this study.
Novel plant genotypes	Plants/plant materials were not involved in this study.
Authentication	Plants/plant materials were not involved in this study.
